# Recent trends in global insecticide use for disease vector control and potential implications for resistance management

**DOI:** 10.1038/s41598-021-03367-9

**Published:** 2021-12-13

**Authors:** Henk van den Berg, Haroldo Sergio da Silva Bezerra, Samira Al-Eryani, Emmanuel Chanda, Bhupender N. Nagpal, Tessa B. Knox, Raman Velayudhan, Rajpal S. Yadav

**Affiliations:** 1 Laboratory of Entomology, Wageningen University, 6700 AA Wageningen, The Netherlands; 2 Department of Communicable Diseases and Environmental Determinants of Health, Pan-American Health Organization/World Health Organization, Washington, DC USA; 3 WHO Regional Office for the Eastern Mediterranean, Cairo, Egypt; 4 WHO Regional Office for Africa, Brazzaville, Republic of Congo; 5 WHO Regional Office for South-East Asia, New Delhi, India; 6 WHO Country Liaison Office, Port Vila, Vanuatu; 7 Department of Control of Neglected Tropical Diseases, World Health Organization, 20 Avenue Appia, 1211 Geneva 27, Switzerland

**Keywords:** Diseases, Infectious diseases, Malaria

## Abstract

Insecticides have played a major role in the prevention, control, and elimination of vector-borne diseases, but insecticide resistance threatens the efficacy of available vector control tools. A global survey was conducted to investigate vector control insecticide use from 2010 to 2019. Out of 140 countries selected as sample for the study, 87 countries responded. Also, data on ex-factory deliveries of insecticide-treated nets (ITNs) were analyzed. Insecticide operational use was highest for control of malaria, followed by dengue, leishmaniasis and Chagas disease. Vector control relied on few insecticide classes with pyrethroids the most used overall. Results indicated that IRS programs have been slow to react to detection of pyrethroid resistance, while proactive resistance management using insecticides with unrelated modes of action was generally weak. The intensive use of recently introduced insecticide products raised concern about product stewardship regarding the preservation of insecticide susceptibility in vector populations. Resistance management was weakest for control of dengue, leishmaniasis or Chagas disease. Therefore, it will be vital that vector control programs coordinate on insecticide procurement, planning, implementation, resistance monitoring, and capacity building. Moreover, increased consideration should be given to alternative vector control tools that prevent the development of insecticide resistance.

## Introduction

Vector-borne diseases cause an unacceptably high burden of mortality and morbidity, with disproportionate effects on the poor and other vulnerable groups. In 2017, vector-borne diseases caused a combined burden of 52 million disability-adjusted life years^1^. Malaria made up the bulk of cases; even after major progress has been reported on malaria control, this progress has recently stalled^2^. Dengue is increasing its geographic distribution and global burden^3^. Other vector-borne diseases, including leishmaniases, Chagas disease, Zika, yellow fever, African trypanosomiasis, onchocerciasis, and schistosomiasis, are less widely distributed than malaria and dengue yet still exact a toll on affected communities.

Vector control has played a major role in the prevention, control, and elimination of several vector-borne diseases^4,5^. The Global Vector Control Response 2017–2030 was launched by the World Health Organization (WHO) as a strategy to enhance implementation of locally-adapted and sustainable vector control as a fundamental approach to preventing disease and responding to outbreaks^6^. Various types and classes of vector control interventions are available or being tested^7^. The mainstay of vector control worldwide has been the use of insecticides to kill or deter vectors. These insecticides have primarily been applied by means of insecticide-treated nets (ITNs), residual spraying, space spraying, larviciding. ITNs include classes of pyrethroid-only nets (long-lasting insecticidal nets and conventionally treated nets), pyrethroid-piperonyl butoxide (PBO, synergist) nets and dual-insecticide nets (adding a pyrrole or a juvenile hormone mimic)^8,9^. In this study, however, we distinguish between ‘factory-treated ITNs’ and ‘ITN-kits’, the latter being insecticide sachets used for periodic, manual treatment of conventionally treated nets by end-users. Residual spraying includes indoor residual spraying (IRS) to kill vectors that land on sprayed interior surfaces as is commonly applied for control of malaria, visceral leishmaniasis and Chagas disease, and outdoor insecticide application in larval habitats and peripheral mosquito resting surfaces, sometimes used in dengue control^10^. Larviciding is the application of insecticides to aquatic habitats to kill mosquito immature stages. Space spraying is the application of cold or thermal fog to cause a short-duration knock-down effect on flying mosquito vectors upon direct contact and has long been the main response to dengue outbreaks despite limited evidence to indicate entomological effect or public health value^11,12^.

The heavy reliance on insecticides for vector control is controversial because of the risk of adverse effects of chemicals on human or animal health and the environment. Moreover, the development of insecticide resistance is a growing problem threatening the continued efficacy of vector control interventions. Malaria vectors have developed widespread resistance to pyrethroids, while resistance to organochlorines, organophosphates, and carbamates has also increased^13,14^. Cross resistance between pyrethroids and the organochlorine dichloro-diphenyl-trichloroethane (DDT) appears to be common in malaria vectors across Africa^15,16^. Resistance has emerged in dengue vectors to all conventional classes of insecticides, particularly to pyrethroids and the organophosphate temephos^17^. Pyrethroid resistance is also a concern in the control of leishmaniasis and Chagas disease vectors^18,19^.

In 2012, WHO published the Global Plan for Insecticide Resistance Management in Malaria Vectors, calling for resistance monitoring systems, and outlining options for rotations, mosaics, combinations, or mixtures to maintain the long-term effectiveness of interventions^20^. As part of the Plan, WHO recommended that insecticides in unrelated classes are ideally rotated annually, and that IRS, if required, should be done with non-pyrethroid insecticides in areas where pyrethroid-ITNs have been distributed. Thus far, no analogous plans have been developed for vector-borne diseases other than malaria. Insecticide resistance management relies on the availability of insecticides with distinct modes of action. Even though 32 modes of action of insecticides have been listed^21^, the available vector control products are based on a few modes of actions, thus limiting options for resistance management. The Innovative Vector Control Consortium (IVCC) was established to overcome barriers in the development of new vector control insecticides^22^. Several vector control insecticide products have recently been brought to market. Notable examples are a micro-encapsulated formulation of pirimiphos-methyl (organophosphate), a polymer-enhanced suspension concentrate of deltamethrin, and two clothianidin-based products (neonicotinoids), for use in IRS; and pyrethroid-PBO nets and dual-insecticide nets^9^. Three modes of insecticidal action are currently recommended for use in IRS and space spraying: acetylcholinesterase inhibitors, voltage-gated sodium channel modulators, and nicotinic acetylcholine receptor competitive modulators. Acetylcholinesterase inhibitors comprise as sub-modes of action carbamates and organophosphates, whilst sodium channel modulators comprise as sub-modes of action pyrethroids and the organochlorine DDT^21^. DDT, the use of which is restricted for disease vector control under the Stockholm Convention on Persistent Organic Pollutants, has not been prequalified by WHO’s new prequalification system.

Retrospective data on insecticide use are valuable for decision making on the use of insecticides for public health and for shaping future strategies of insecticide resistance management. The objective of this study was to investigate patterns in insecticide use for vector control in public health during the past decade, with respect to amounts used, insecticide classes, intervention types, and diseases targeted, and to examine the implications of observed patterns for insecticide resistance management and, ultimately, for sustainable disease control. We refer to insecticide use in terms of spray coverage, rather than physical amounts, to allow for comparison between insecticide active ingredients and among intervention types. Further evaluation of data by physical amounts of active ingredient deployed is available in a separate report^23^.

## Results

### Survey response and sample selection

**Figure 1:**
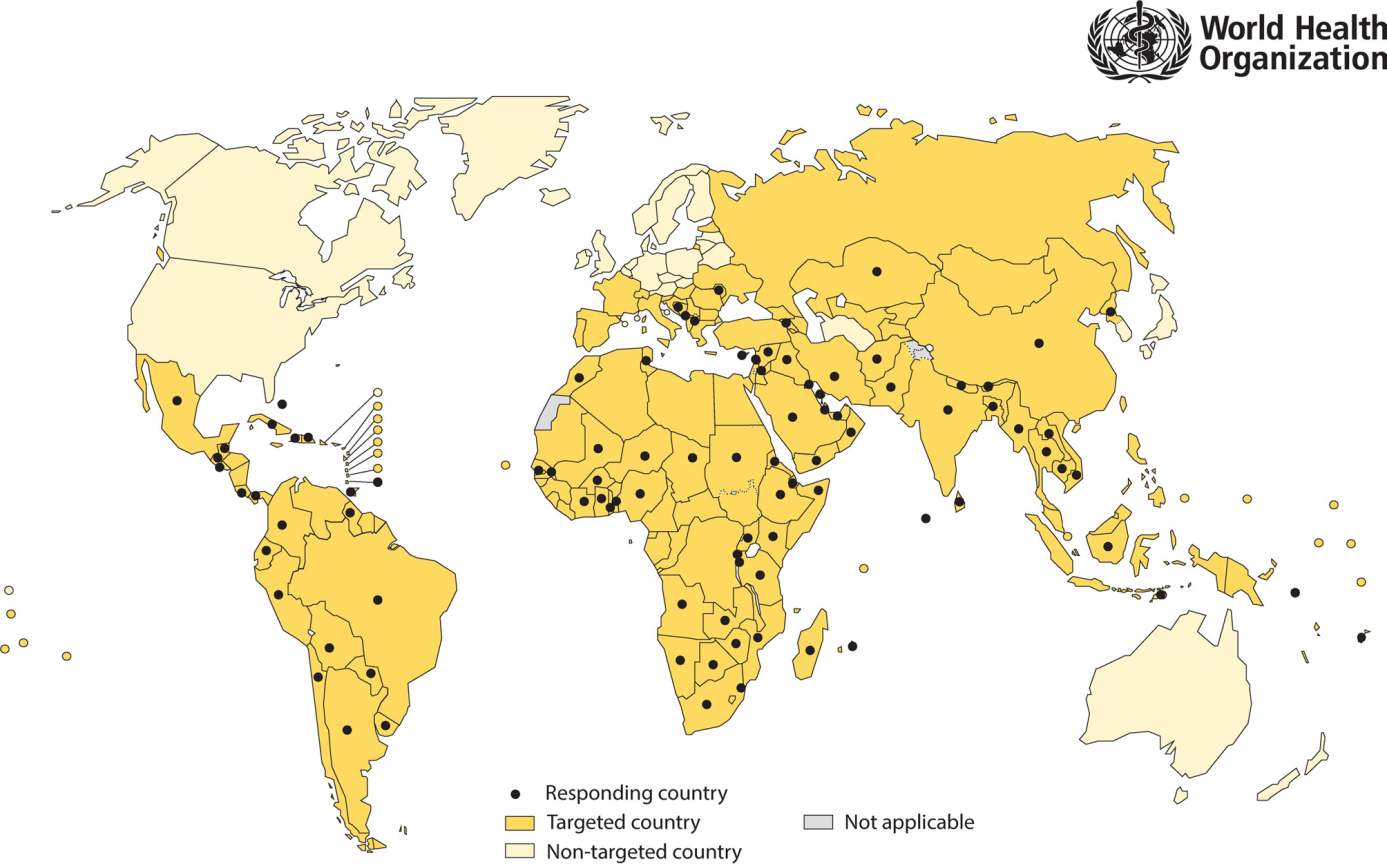
Map showing targeted and responding countries. The boundaries and names shown, and the designations used on this map do not imply the expression of any opinion whatsoever on the part of the World Health Organization concerning the legal status of any country, territory, city, or area or of its authorities, or concerning the delimitation of its frontiers or boundaries. Dotted lines on maps represent approximate border lines for which there may not yet be full agreement. Data source: World Health Organization (WHO). Map production: Control of Neglected Tropical Diseases, WHO. ^©^WHO 2021. All rights reserved. Written permission to use and adapt the map was granted by WHO.

At the outset, 164 countries from 5 United Nations regions were targeted for the study, out of which 98 countries responded and 92 countries provided data on insecticide use for one or more years from the period 2010–2019 (Fig. 1). However, the response from two United Nations regions was low or absent; from the Eastern European Region, 5 out of 16 targeted countries responded (representing 4.8% of the targeted population) whilst from the Western European & Others Region, 0 out of 8 targeted countries responded. Because of their unsatisfactory response, and because of being relatively free from mosquito-borne diseases, the Eastern European Region and the Western European & Others Region were excluded from the sample of our study. Out of the remaining 140 countries that were selected for our study, 87 countries provided insecticide use data (62.1% country response rate); these countries comprised 33 African, 32 Asia–Pacific and 22 Latin American & Caribbean countries (Supplementary Table S1). The annual response rate adjusted for population size, which was used as the weighting factor in the analysis, was 76.5–87.4% (average 83.5%) for 2010–2019 and was lowest for the more recent years (Supplementary Table S1).

### Insecticide use

The diseases with highest use of insecticides in spraying operations (i.e., residual spraying, space spraying, larviciding), in terms of ‘standard spray coverage’ (see “Methods”), were malaria (60.8% of total use, pooled over regions and years), dengue (22.9%), leishmaniasis (9.7%) and Chagas disease (4.8%). In addition, insecticides were used in ITNs for malaria control. These four diseases became the focus of our analysis; other vector-borne diseases, which jointly accounted for only 1.8% of vector control insecticide use, were excluded.

The weighted annual amounts of insecticides in spraying operations in the selected countries, expressed in active ingredient per insecticide class, and pooled over diseases, regions, and years, were 3042 t of organochlorines, 1489 t of organophosphates, 611 t of carbamates, 174 t of pyrethroids, 33 t of neonicotinoids, 77 t of bacterial larvicides, 18 t of insect growth regulators, and 14 t of spinosyns. The ten most used insecticide active ingredients in spraying operations in terms of standard spray coverage (pooled over diseases, regions, and years), were the organochlorine DDT (21.7% of total use); the pyrethroids deltamethrin (20.1%), alpha-cypermethrin (13.4%) and lambda-cyhalothrin (11.8%); the carbamate bendiocarb (7.6%); the organophosphates malathion (4.6%) and pirimiphos-methyl (4.0%); the insect growth regulator pyriproxyfen (3.3%); the organophosphate temephos (3.0%); and the carbamate propoxur (2.3%) (Supplementary Table S2).

### Insecticide use, by intervention type

**Figure 2:**
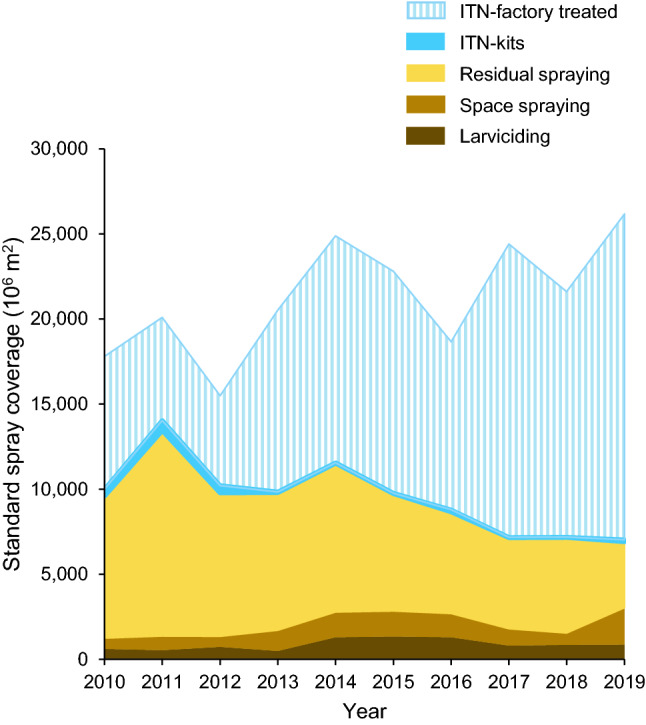
Area graph of global use of vector control insecticides by intervention type. Results are pooled for the four selected diseases and are expressed in standard spray coverage.

Factory-treated ITNs were the intervention type that made a 55% contribution to global vector control insecticide use in terms of ‘standard spray coverage’ (pooled over regions, diseases, and years), and its annual share increased from 43.8% in 2010 to 73.1% in 2019 (Fig. 2; Supplementary Table S3). Fluctuations in ITNs are probably related to donor-driven deliveries. Global use of ITN-kits was minor (0.9%), largely reported from Cambodia, China and Vietnam, and its annual global share declined from 2.4% in 2010 to 0.5% in 2019. The contribution of residual spraying to vector control insecticide use was 34.5% and its annual share fell from 46.8% in 2010 to 14.8% in 2019; residual spraying was for 97.2% by IRS, the remaining 2.8% being for outdoor perifocal treatment. The peaks in residual spraying in 2011 and 2014 were mainly attributable to high use in one country (Mexico). Space spraying had an overall contribution of 5.5% and showed an increase in the year 2019. The contribution of larviciding was 4.1% and did not show a trend.

### Insecticide use, by class

**Figure 3:**
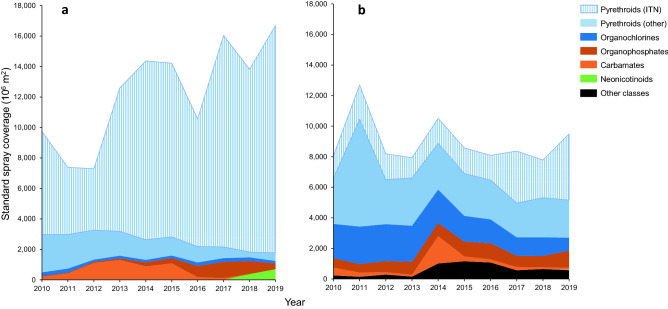
Area graph of vector control insecticide use by insecticide class. (**a**) African Region, (**b**) Asia–Pacific Region plus Latin American & Caribbean Region combined. Results are pooled for the four selected diseases and expressed in standard spray coverage. Striped pattern indicates use of insecticides in ITNs; non-striped pattern indicates use in spraying operations (i.e., residual spraying, space spraying, larviciding). Shades of blue or shades of red indicate insecticide sub-groups within a mode of action^21^. ‘Other classes’ represent bacterial larvicides, insect growth regulators, spinosyns, and pyrroles.

In the African Region, the use of pyrethroids in ITNs, residual spraying and space spraying accounted for 89.9% of all vector control insecticide use in terms of ‘standard spray coverage’ (pooled over diseases and years), indicating a dominance of this insecticide class, with an annual share of 81.8–94.9% (Fig. 3a; Supplementary Table S4). Pyrethroid use in ITNs alone made a 79.0% contribution to insecticide use, increasing from 69.5% in 2010 to 89.6% in 2019, whilst pyrethroid use in residual and space spraying operations made a 11.0% contribution to insecticide use, decreasing from 25.4% in 2010 to 3.2% in 2019. Pyrethroids were not reported, nor recommended, for use in larviciding. The contribution of organochlorines (average 1.7%; 1.0–3.6%), organophosphates (average 3.1%; 0.4–6.9%), carbamates (average 4.4%; 0–15.4%), neonicotinoids (average 0.9%; 0–4.0%) and other classes (average > 0.1%) was relatively small in the African Region.

In Asia–Pacific and Latin American & Caribbean Regions, pyrethroid use in ITNs and in residual and space spraying operations combined accounted for 58.8% of vector control insecticide use (pooled over diseases and years), increasing from 52.8% in 2010 to 71.1% in 2019 (Fig. 3b; Supplementary Table S4). The contribution of pyrethroids in ITNs alone was 22.7%, increasing from 13.6% in 2010 to 44.9% in 2019, whilst the contribution of pyrethroids in residual and space spraying operations was 36.1%, decreasing from 39.2% in 2010 to 26.2% in 2019. The contribution of organochlorines was 20.8%, decreasing from 29.3% in 2010 to 9.1% in 2019. Smaller contributions were made by organophosphates (average 9.2%; 4.5–13.2%), carbamates (average 4.5%; 1.5–17.2%), neonicotinoids (average 0.1%; 0–0.5%) and other insecticide classes (average 6.7%; 1.2–13.6%). The ‘other’ insecticide classes consisted of an average of 60.5% insect growth regulators, 22.6% spinosyns, and 16.9% bacterial larvicides in the Asia–Pacific and Latin American & Caribbean Regions.

The use of the organochlorine DDT at global level decreased by 62.0% during the study period, with 13 countries reporting DDT use in 2010 and five countries still using DDT in 2019.

### Insecticide use, by disease

**Figure 4:**
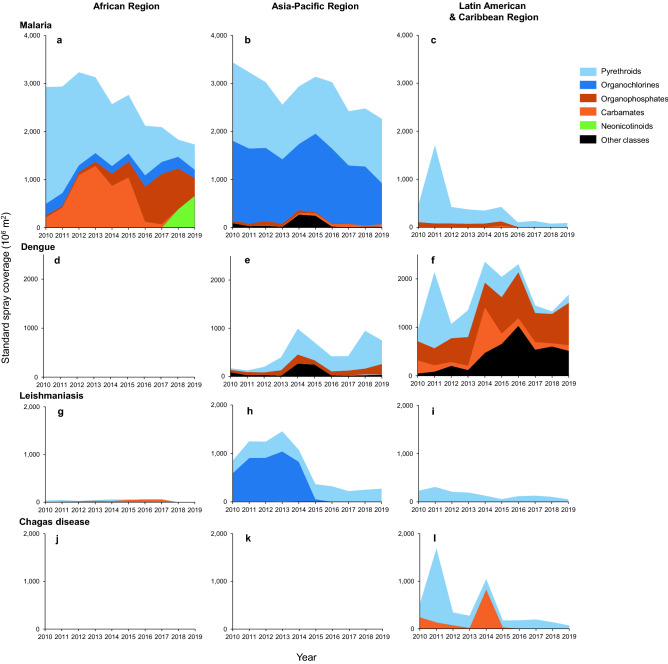
Area graph of vector control insecticide use, excluding use in ITNs, by disease. Results are presented for three geopolitical regions, (**a**–**c**) for control of malaria, (**d**–**f**) dengue, (**g**–**i**) leishmaniasis, and (**j**–**l**) Chagas disease, and expressed in standard spray coverage. Shades of blue or shades of red indicate insecticide sub-groups within a mode of action^21^. ‘Other classes’ represent bacterial larvicides, insect growth regulators and spinosyns (all used as larvicides).

For each of the four selected diseases, regional results are presented on insecticide use in spraying operations, which excludes ITNs (Fig. 4; Supplementary Table S5).

Insecticide use for control of malaria in the African Region, which was for 98.7% by IRS and for 1.0% by larviciding, declined by 40.8% during the study period. The pattern of use exhibited clear temporal shifts between insecticide classes (Fig. 4a). Use of pyrethroids (i.e., for 53.2% deltamethrin and 43.7% lambda-cyhalothrin, pooled over years) was highest in 2010, but by 2012, pyrethroids had been partly replaced by carbamates (i.e., for 79.9% bendiocarb and 20.1% propoxur, pooled over years). From 2014, the contribution of carbamates and pyrethroids declined but that of organophosphates (i.e., for 93.5% pirimiphos-methyl and 6.5% temephos, pooled over years) increased. Four African countries that had shifted to a new formulation of pirimiphos-methyl relied exclusively on this insecticide for IRS for four to six consecutive years. From 2018, the contribution of organophosphates declined while that of neonicotinoids (clothianidin as only active ingredient used) increased. In 2019, neonicotinoids were the most common insecticide class used for malaria control in the African Region (38.5% of total use in 2019). Pyrethroids made a partial comeback in Africa with a 49.1% increase from 2018 to 2019, which was entirely attributable to the use of Fludora^®^ Fusion, a new combination product with clothianidin and deltamethrin. The use of organochlorines in Africa (DDT as the only active ingredient) was relatively small and rather constant over the years (4.5–13.2% of annual use). The use of insecticides in other classes, all of which were larvicides, was negligible in Africa (0.02% of total use).

In the Asia–Pacific Region, insecticide use for malaria control, which was for 92.7% by IRS, 4.4% space spraying, and 3.0% larviciding (pooled over years), declined by 34.3% during the study period. Unlike in the African Region, the pattern of use in the Asia–Pacific did not reveal a clear temporal shift between insecticide classes (Fig. 4b). Instead, pyrethroids (i.e., for 55.7% alpha-cypermethrin, 25.3% deltamethrin and 19.0% lambda-cyhalothrin; pooled over years) and organochlorines (DDT as only active ingredient and reported only from India) remained the two main insecticide classes throughout the study period. Unlike in Africa, uses of carbamates, organophosphates and neonicotinoids were minimal in the Asia–Pacific. In the third region, the Latin American & Caribbean, insecticide use for malaria control was low relative to the other regions, displaying a declining trend, and was largely composed of pyrethroids and organophosphates (Fig. 4c).

Insecticide use for control of dengue was negligible in the African Region (Fig. 4d). In the Asia–Pacific, reported insecticide use for dengue—which was for 40.4% by residual spraying, 33.2% larviciding and 26.4% space spraying (pooled over years)—exhibited a fluctuating pattern; insecticide use for vector adulticiding (i.e., residual spraying and space spraying) was for 94.4% by pyrethroids (pooled over years), while organophosphates were mostly used for larviciding (i.e., 85.2% of organophosphates; pooled over years) (Fig. 4e). Insecticide use for dengue was highest in the Latin American & Caribbean Region, where 46.3% of insecticides was used for space spraying, 34.3% for larviciding, and 19.5% for residual spraying (pooled over years). Insecticide use for dengue in this region was on average 3.25 times higher than in the Asia–Pacific even though the total population of the region was only 14% that of the Asia–Pacific. This signifies a high per-capita insecticide use for dengue control in the Latin American & Caribbean Region. In this region, pyrethroids and carbamates had gradually been replaced with organophosphates, whilst other insecticide classes, primarily spinosyns and bacterial larvicides, had a variable contribution (Fig. 4f). In the Latin American & Caribbean Region, pyrethroids peaked in 2011, which was due to high reported use against multiple vector-borne diseases in one country (Mexico). Insecticide use for dengue control may have been under-estimated because many countries have decentralized dengue control programs, which likely reduced availability of insecticide use data at central level. Also, insecticides procured and reported for malaria control may in part be deployed for emergencies of other diseases such as dengue.

Insecticide use for control of leishmaniasis was substantial only in the Asia–Pacific Region, where it was for 98.0% by IRS (Fig. 4g–i). In the Asia–Pacific, the use of the organochlorine DDT was reported from one country, India, for control of visceral leishmaniasis; after DDT use against this disease was completely stopped after 2016, only pyrethroids were used (Fig. 4h). Insecticide use for leishmaniasis control was lower than for malaria or dengue control, in part because of a lower global burden of leishmaniasis.

Insecticide use against Chagas disease, a disease with a relatively low global burden, was reported only from the Latin American & Caribbean Region and was for 95.7% by IRS (Fig. 4j–l). The reported use in recent years has been low, apart from peaks in the use of pyrethroids in 2011 and carbamates in 2014 (Fig. 4l).

### Insecticide resistance management

The percentage of countries with confirmed pyrethroid resistance in malaria vectors in a particular year, and with pyrethroids in use for IRS in that year, did not decline significantly from 2010–2014 to 2015–2019 (Table 1; Supplementary Table S6). This suggests that many countries continued using pyrethroids in IRS for malaria control despite detected resistance. Conversely, countries with confirmed resistance but without pyrethroids in use increased significantly from 2010–2014 to 2015–2019, indicating that some countries with pyrethroid resistance stopped using pyrethroids in IRS. Hence, many countries did not react to detection of pyrethroid resistance during the study period, whereas others switched to other insecticide classes after several years. The number of countries eligible for analysis decreased over the study period, possibly because of a delay in reporting or because the number of countries using IRS for malaria control declined from 49.2 in 2010–2014 to 38.4 in 2015–2019. Some African countries with detected pyrethroid resistance, notably Benin, Burkina Faso, and Uganda, used only non-pyrethroids for IRS over the periods 2010–2014 and 2015–2019. Examples of countries that had switched from pyrethroids to non-pyrethroids in the period 2015–2019 were Senegal, Tanzania, and Zambia. Several Asia–Pacific countries continued relying on pyrethroids even after resistance was detected whilst the alternative compounds of pirimiphos-methyl and clothianidin had not been introduced. A limitation of using country-level data is that within-country variations are not accounted for. For example, a country may have detected pyrethroid resistance in one region but have pyrethroids in use in another region.

**Table 1:** Pyrethroid resistance in relation to pyrethroid use per country.

Four categories of conditions^a^			Number of eligible countries, by year										% per category		*P*^*b*^
Category	Confirmed resistance	Pyrethroids in use	2010	2011	2012	2013	2014	2015	2016	2017	2018	2019	2010–2014	2015–2019	
1	Yes	Yes	7	8	6	9	8	4	7	5	3	1	34.2%	27.4%	*n.s*
2	Yes	No	3	3	6	5	7	9	9	5	3	6	21.6%	43.8%	0.002
3	No	Yes	10	8	12	7	9	9	2	3	4	2	41.4%	27.4%	0.05
4	No	No	0	0	0	3	0	1	0	0	0	0	2.7%	1.4%	*n.s*
Sum			20	19	24	24	24	23	18	13	10	9	100.0%	100.0%	

Presented is the number of eligible countries in each category per year. ‘Confirmed resistance’ refers to < 90% mortality of anophelines in insecticide susceptibility bioassays for pyrethroids^24^. ‘Pyrethroids in use’ refers to use in IRS for malaria control.

^a^Categories: 1, confirmed resistance, pyrethroids in use; 2, confirmed resistance, no pyrethroids in use; 3, no confirmed resistance, pyrethroids in use; 4, no confirmed resistance, no pyrethroids in use.

^b^χ^2^ test (*df* = 1) between the two periods for each category; *n.s*., not significant (*P* > 0.05).

The degree of proactive insecticide resistance management for disease vector control, as measured by use patterns of insecticides with multiple modes of action, was highest in the African region for control of malaria but did not display a significant improvement from 2010–2014 to 2015–2019 (Table 2; Supplementary Table S7). Countries with highest *R-*values (see “Methods”) in 2015–2019 were Botswana (*R* = 4.8), Eswatini (*R* = 8.3), Madagascar (*R* = 5.6), Mozambique (*R* = 6.6), Zambia (*R* = 5.4) and Zimbabwe (*R* = 5.5); these countries had made notable progress in resistance management from 2010–2014, except for Mozambique, which had a high *R-*value in both periods. As a benchmark, a country using a single mode of action year after year attains an *R*-value of 0.0, whilst a country that rotates annually between three modes of action (i.e., each being used once every 3 years, which could be considered as a desirable strategy) would attain an *R-*value of 10.0 over a 5-year period. *R-*values were low in the Asia–Pacific Region for malaria, dengue and leishmaniasis (Table 2). In the period 2015–2019, seven out of nine Asia–Pacific countries had an *R-*value of zero for malaria vector control, because pyrethroids were the only class used. The Latin America & Caribbean region also showed low *R-*values for malaria, dengue, and Chagas disease, suggesting a lack of proactive resistance management (Table 2).

**Table 2:** Degree of proactive resistance management for disease vector control.

Geopolitical region	Vector-borne disease	Period 1: 2010–2014				Period 2: 2015–2019				*N*	*P*^*a*^
		Rotation	Mosaic/combi-nation	Multiplicity	Sum (*R*)	Rotation	Mosaic/combi-nation	Multiplicity	Sum (*R*)		
African	Malaria	1.41	0.55	1.03	2.99	1.79	0.46	1.35	3.62	17	*n.s*
Asia–Pacific	Malaria	0.67	0.33	0.56	1.56	0.11	0.19	0.39	0.69	9	*n.s*
	Dengue	0.36	0.60	0.64	1.60	0.36	0.57	0.64	1.57	7	*n.s*
	Leishmaniasis	0.20	0.24	0.40	0.84	0.00	0.20	0.20	0.40	5	*n.s*
Latin American & Caribbean	Malaria	0.14	0.17	0.29	0.60	0.29	0.29	0.43	1.00	7	*n.s*
	Dengue	0.14	0.63	0.64	1.41	0.21	0.86	0.93	2.00	7	*n.s*
	Chagas disease	0.00	0.33	0.33	0.67	0.00	0.33	0.33	0.67	3	*n.s*

Values are shown of parameter *R* and its components of rotation, mosaic/combination spraying, and multiplicity of modes of action, in eligible countries (*N*) during two 5-year periods.

*n.s*., not significant (*P* > 0.05).

^a^Paired t-test (two-tailed) of *R*-values between the two periods.

## Discussion

A central problem in disease vector control has been its reliance on few insecticide classes and, hence, its vulnerability to insecticide resistance^14^. This problem emphasizes the importance of developing alternative paths towards achieving rational and sustainable vector control. Evidently, pyrethroids were the dominant insecticide class used in vector control spraying operations, in terms of spray coverage, even though pyrethroid use in IRS substantially declined in the African Region in association with the detection of widespread pyrethroid resistance in malaria vectors. Organochlorines, with DDT as only active ingredient, were the second most used insecticide class in terms of spray coverage; the global use of DDT declined steadily during the past decade, possibly due to policy change in response to detected resistance, availability of alternative insecticides, or the effect of the Stockholm Convention as legally binding instrument. The dependence on few insecticide classes was evident in the Asia–Pacific and Latin American & Caribbean Regions, whilst in the African Region, a new insecticide class, neonicotinoids, had recently been added for malaria vector control.

With the inclusion of ITNs in insecticide use data, the dominance of pyrethroids was enormous. ITNs have had a major contribution to vector control insecticide use, particularly in Africa, where many countries use ITNs as only malaria vector control tool^25^. The global contribution of ITNs has gradually increased while that of IRS has decreased. In Africa, the decline in IRS was partly because new IRS products were costlier than the pyrethroids they replaced, leading to a lower coverage of IRS because of limited resources^26^. In spite of widespread pyrethroid resistance, ITNs have continued to provide protection against malaria transmission^27^, while pyrethroid-PBO nets have demonstrated improved control over pyrethroid-only nets where pyrethroid resistance was present^28,29^. Disease vectors will persist, however, in adapting to interventions, owing to the mass killing effect of ITNs on vector populations^30^, which exerts selection pressure for insecticide resistance. In this respect, a study from Mozambique reported extensive loss in efficacy of a pyrethroid-PBO net against *An. funestus*^31^. Dual-insecticide ITNs add a new mode of action to the ITN-intervention type but, so long as non-pyrethroid ITNs have not been developed and marketed, vector populations will continue to be exposed to pyrethroids. If resistance alleles are allowed to increase to high frequencies under constant selection pressure over time, resistance could become ‘fixed’ in a vector population^15^. Unless drastic measures are taken, pyrethroids may lose their value as an insecticide class for protection of public health^13^.

Our findings indicated that many countries have been slow to react to detected pyrethroid resistance in malaria vectors by delayed switching to non-pyrethroid insecticides for IRS. Moreover, we presented evidence that proactive resistance management in malaria vectors, through rotations, mosaics, or combinations of insecticides with unrelated modes of action, was generally poor, with exception of several African countries, and did not significantly improve over the 10-year study period. A recent study made a similar observation^25^. There was no evidence of implementation of resistance management in the Asia–Pacific and Latin American & Caribbean, including for malaria, dengue and leishmaniasis control. Plausible reasons for poor resistance management are (1) the absence of a national resistance management plan; (2) weak resistance monitoring systems and databases; or (3) limited access to insecticides in multiple classes. The number of African countries with a national plan for insecticide resistance management reportedly increased from seven in 2014 to eighteen in 2018^32,33^, but less progress was reported from other regions^33^. A resistance monitoring system was reported from half of African countries; again, there was less evidence of progress in other regions^34^.

In the past decade, new insecticide products have been swiftly adopted for IRS by malaria control programs. The risk of complacency in this regard is evident in examples from the agricultural sector^35^. The sequential pattern of use of different insecticide classes observed in Africa, with a period of pyrethroid dominance followed by periods of intensive use of carbamates, organophosphates, and, most recently, neonicotinoids, suggests that a proactive strategy of frequent rotation of classes to manage resistance has generally not been implemented. The pattern was reportedly driven by national-level policy change in response to detected resistance in bioassays, or by the availability of superior alternatives products^26^, and possibly also by development partner contributions to subsidize newer and costlier products. For example, bendiocarb was after several years of intensive use replaced with pirimiphos-methyl because of reports of bendiocarb resistance and superior residual period of pirimiphos-methyl^26^. Similarly, we found that several African countries which adopted pirimiphos-methyl used it over consecutive years as ‘monotherapy’, until clothianidin became available. The prolonged periods of intensive use of singular products were likely a strong selector for resistance development. In retrospect, this pattern did not display optimal product stewardship, even as countries and programs had little choice of using several insecticide classes in annual rotations or in mosaics. Along the same line, the new product Fludora^®^ Fusion containing clothianidin plus deltamethrin has been adopted for IRS by countries but available guidance by WHO recommends that vectors should be susceptible to the insecticides being deployed in IRS, which implies that the mixture containing a pyrethroids should not be used in areas where pyrethroid resistance has been detected. Considering the costly and lengthy process of product development^22^, the introduction of new products or product portfolios should come with a long-term plan for stewardship commitment by manufacturers, programs and donors aiming to preserve insecticide susceptibility in vector populations^14,36^.

Unfortunately, the knowledge base to inform decision making on resistance management in disease vectors is fragmented at best^37,38^, although efforts have been made to systematically compile and present insecticide resistance data at global level, especially for malaria^24^. In theory, rotations reduce the selection of resistance alleles if periods of use are sufficiently short and restore insecticide susceptibility if periods of non-use of a particular insecticide are sufficiently long^35^. However, the speed of resistance development and reversal to susceptibility depends on variables including the initial frequency of resistance alleles, the intensity of insecticide use (including from agricultural and domestic insecticide uses), fitness cost in vector species, the resistance mechanism, and immigration of susceptible vectors^39,40^. Pyrethroid resistance in malaria vectors across sub-Saharan Africa increased dramatically between 2005 and 2017^41^, and in some countries within three years of ITN or IRS campaigns^42^. Laboratory and field evidence suggests that, in most cases, the frequency of resistance alleles decreased after the selector was removed, but reversion rates were variable^43^. As some countries have stopped using pyrethroids in IRS for malaria control, this is an opportunity to monitor reversion rates in field settings. As an indication, in Zambia, a substantial reduction in deltamethrin resistance in *An. funestus* was reported two years after pyrethroids had been replaced by pirimiphos-methyl^44^.

In practice, proactive resistance management can be a point of contention with national or donor agencies. For example, a switch to a costlier insecticide could be unacceptable in short-term decision making but optimal in a longer-term strategy that values insecticide susceptibility as public good that should be preserved^37^. Moreover, having multiple insecticides available for use in rotational or mosaic strategies inevitably puts logistic demands on the system of registration, procurement, safety precautions, stock management, and operational use, and likely at additional cost. Such challenges underscore the importance of a nationally coordinated plan for insecticide resistance which is supported by relevant national institutions.

Larviciding has only made a minor contribution to overall global use of vector control insecticides but deserves increased attention in the context of insecticide resistance management because unique modes of action are available for larviciding that are not available for vector adulticiding^9,32^. Moreover, WHO’s Global Vector Control Response 2017–2030 called for new tools, technologies and approaches to counter insecticide resistance and other challenges^6^. Non-insecticidal approaches such as house improvement and environmental management could, where appropriate, contribute to vector control without causing resistance. In dengue control, the elimination of breeding sites has been advocated as mainstay vector control^45^, but the scale of implementation requires investigation. The role of non-chemical approaches in integrated vector management strategies deserves increased attention at all levels.

Malaria programs have benefited from the Global Plan for Insecticide Resistance Management through the development and implementation of national plans^32,44^. However, our data suggest that insecticide resistance management was weakest for control of dengue, leishmaniasis and Chagas disease, despite evidence of resistance in vector populations, whilst entomological expertise has commonly been concentrated within malaria programs^46^. Hence, the Plan should include other vector-borne diseases^44,47^, with monitoring procedures adapted to those vectors^48^, and coordination between vector control programs on insecticide procurement, planning, implementation, and resistance monitoring. These activities must be supported by adequate capacity building of public health entomologists. Also, coordination on insecticide resistance management between programs and sectors within countries is vital. Agricultural insecticide use is known to accelerate the resistance development in malaria vectors, particularly in commercial crops^49^. Recent data from Cameroon indicated pre-existing resistance of a malaria vector to the neonicotinoid clothianidin in association with agricultural use^50^, which is worrisome considering that neonicotinoids have acquired a major agrochemicals market share^21^. Coordinated resistance management between health and agriculture will have potential benefits for the outcomes in both sectors. Moreover, household aerosol insecticide products can contribute to selection of pyrethroid resistance in disease vectors^51^. Hence, the role of agricultural and household insecticides should be factored into resistance management strategies, supported by legislation on their use and purpose of use, and building intersectoral linkages to adopt, as appropriate, legal restrictions on the use of insecticide classes, and integrated strategies for management of pests and vectors^33,52^.

## Methods

### Data collection

An electronic spreadsheet form, in English, French and Spanish, was used to solicit annual insecticide use data from targeted countries over the period 2010–2019; the form contained the following columns: ‘year’, ‘compound or product’, ‘class’, ‘formulation’, ‘concentration’, ‘type of application’, ‘for control of’, ‘amount of formulation used (kg or L)’, ‘amount of active ingredient used (kg or L)’, and ‘comments, if any’. Explanatory notes were provided. WHO Member States were targeted which were at significant risk or had a significant burden of vector-borne diseases (Fig. 1). The reporting form was sent from WHO’s Regional Offices via WHO Country Offices to the national focal point in the Ministry of Health for completion by the manager of the main national vector-borne disease control programme, or by the national manager of entomological surveillance and vector control. The survey was first conducted in 2015, to cover data from 2010 to 2014, and again in 2020, to cover data from 2010 to 2019.

Data on the number of factory-treated ITNs delivered were extracted by J. Milliner (pers. comm., 2020) from a dataset compiled by the Net Mapping Project^53^. The content of insecticide active ingredient and synergist per m^2^ of net fabric for each ITN product were obtained from WHO specifications^9^, and the amounts of active ingredient and synergist (in g per ITN) were calculated per product based on a net size of 1.9 m length, 1.8 m width and 1.5 m height (14.52 m^2^ of fabric per ITN). Data for individual products were subsequently anonymized to maintain commercial confidentiality, by pooling the data per active ingredient, and were then provided by J. Milliner to WHO. The data differentiated between sub-Saharan Africa and ex-Africa. To present the results according to United Nations Regional Groups^54^, adjustments were made based on ITNs delivered in individual countries in the period 2010–2019^53^. Hence, the results for sub-Saharan Africa were equivalent to those for the African Region because annual ITN deliveries in North Africa had been negligible (0.01%) relative to those in sub-Saharan Africa. Results for ex-Africa were equivalent to those for the Asia–Pacific plus Latin American & Caribbean regions only after annual results had been deflated by 1.35% per year, on average, to exclude the share of the Eastern European and Western European & Others regions. Use of factory-treated ITNs was assumed to be for control of malaria, but minor uses for control of other diseases may have taken place.

### Estimation of spray coverage

The comparison between different active ingredients is complicated by differences in spray utility, which determines the dosage at which an active ingredient is applied to surfaces, spaces or water bodies^55^. Notably, most pyrethroids are very potent insecticides, recommended for use at application rates 1/60th that of many non-pyrethroid insecticides. Moreover, the comparison among intervention types is complicated by differences in area units. Application rates have been expressed in g/m^2^ of surface for IRS; in g/ha for space spraying and larviciding; or in g/m^2^ of netting fabric for ITN. To enable comparison between active ingredients, and among intervention types, a proxy of insecticidal action was provided by the ‘standard spray coverage’, defined as the surface covered by a given amount of active ingredient as for use in a single application of IRS, and assuming operations complied with internationally recommended application rates. Hence, standard spray coverage (m^2^) was calculated as the amount of active ingredient (g) used divided by the recommended application rate as for use in IRS (g/m^2^). Data on application rates per active ingredient are presented in the “Supplementary Methods”.

### Data processing and analysis

The survey data were entered into an electronic spreadsheet (Dataset S1). The amounts of formulated insecticide product were converted to g of active ingredient using the provided concentrations, and assuming 1 mL of liquid product weighs 1 g. For bacterial larvicide *Bacillus thuringiensis israelensis*, the concentration of formulated products were derived from the only available reference standard^56^. The amount of active ingredient (g) of an insecticide product was divided by the spray utility (g/m^2^) to yield the standard spray coverage (m^2^). Where an active ingredient was reported to be used against two or more diseases, without specifying the amounts used per disease, it was assumed that equal amounts were spent per disease; this was a reasonable assumption considering that the use against multiple diseases constituted a minority (21%) of the total insecticide spray coverage. The scope of analysis was limited to those vector-borne diseases against which most insecticides were found to be used. Excluded from analyses were uses of repellents for application on human skin and mineral oils for larviciding, both sporadically reported by countries; no WHO specifications were available for mineral oils. For comparison of results between geopolitical regions, referred to as regions, countries were grouped according to the United Nations Regional Groups of Member States^54^, which are: the African, Asia–Pacific, Latin American & Caribbean, Eastern European, and Western European & Others groups of countries. This grouping differed from WHO regions; for example, countries of the WHO Eastern Mediterranean Region were allocated to the African and Asia–Pacific Regional Groups for our analysis. To adjust for missing data from individual countries for certain years, annual data on insecticide use at regional or global level were divided by an annual weighting factor. Because insecticide use was assumed to be dependent on a country’s population size, the weighting factor in year *y* was calculated by dividing the total population of countries that responded in year *y* by the total population of targeted countries; this was done for the sample of countries that were selected for analysis. The annual weighting factor was also applied to calculations of data pooled over years. Population data for 2019 were used^57^.

### Assessment of insecticide resistance management

Resistance management can use a reactive or proactive approach. Reactive resistance management is defined here as the switch to another insecticide in response to detected resistance, whilst proactive resistance management, in the narrow sense (i.e., excluding non-insecticidal intervention types), is defined here as the use of insecticides with multiple modes of insecticidal action in rotations, mosaics, combinations, or mixtures, intended to prevent resistance development.

To study reactive resistance management, two data types were paired per country per year: publicly available data on insecticide susceptibility, and insecticide use data. The scope of the analysis was limited to: (1) malaria vectors, for which adequate susceptibility data were publicly available; (2) pyrethroids, which is the insecticide class most compromised by resistance development; and (3) the study period 2010–2019. Details about pairing, eligibility and categorization of data are provided in the “Supplementary Methods”. Four categories of conditions were: (1) confirmed resistance, pyrethroids in use; (2) confirmed resistance, no pyrethroids in use; (3) no confirmed resistance, pyrethroids in use; (4) no confirmed resistance, no pyrethroids in use. The number of countries in each category was related to the sum of countries in all categories. A χ^2^*-*test was used to test for differences in the contribution of each category between the two data collection periods, 2010–2014 and 2015–2019.

To study proactive resistance management, the annual patterns and diversity of insecticide classes were examined for individual countries. Over the years, scientists have wrestled with the problem of quantifying resistance management and to date there is no simple equation available. In a modest attempt to overcome this bottleneck, we developed Eq. (1) for resistance management (*R*) in vector adulticiding (i.e., residual spraying, space spraying) in individual countries over a selected period of *p* years:

1 $$R = \sum\limits_{x = 1}^{p} {\left( {a_{x} } \right) + \left( {b - 1} \right) + \left( {c - 1} \right)}$$

whereby *a* denotes the degree of insecticide rotation, calculated as the number of modes of action added or removed in each subsequent year over period *p* (score of 1 per mode of action added or removed; score of 0.5 per sub-mode of action); *b* the degree of mosaic or combination spraying, calculated as the average number of modes of action used per year over *p* years; and *c* the multiplicity of modes of action over time, calculated as the total number of modes of action used over period *p*. A five-year period (*p* = 5) was selected to allow for examination of a rotational pattern, while also permitting comparison between two periods within the ten-year study period, namely 2010–2014 and 2015–2019. Modes of action were specified according to an available classification scheme^21^. Equation (1) has several limitations: it assumes equal importance to *a*, *b,* and *c*; mosaics or combinations cannot be differentiated from rotations at sub-national level; moreover, the assumption that a switch between modes of action (e.g., from pyrethroids to organophosphates) has double the effect than a switch between sub-modes of action (e.g., from pyrethroids to organochlorines) does not account for the resistance mechanism. Larvicides were excluded from analysis because of small average amounts used by countries. Countries eligible for analysis were those reporting disease-specific insecticide use data in at least 9 years over the 10-year period 2010–2019. A paired t*-*test (two-tailed) was used for differences in *R-*values between the two periods.

## Supplementary Information

Supplementary Information.

## Data Availability

The country response list and data synthesis are included in the article and the Supplementary Information. The survey dataset generated during the study is available from the corresponding author on reasonable request.
