# Supplementary material for: Recent trends in global insecticide use for disease vector control and potential implications for resistance management
Source: Sci Rep. 2021 Dec 13;11:23867. doi: 10.1038/s41598-021-03367-9 (PMC8669011; doi:10.1038/s41598-021-03367-9)
Supplement: Supplementary file 1 — Supplementary Information. [file 41598_2021_3367_MOESM1_ESM.pdf]

# Supplementary Information

## Recent trends in global insecticide use for disease vector control and potential implications for resistance management

Henk van den Berg, Haroldo Sergio da Silva Bezerra, Samira Al-Eryani, Emmanuel Chanda, Bhupender N. Nagpal, Tessa B. Knox, Raman Velayudhan, Rajpal S. Yadav

**This file includes:**

Supplementary Methods.

Supplementary Notes

Supplementary Table S1. Targeted countries, with population size and annual responses, and calculation of annual weighting factor.

Supplementary Table S2. Use of active ingredients for vector control spraying operations in ranking order (pooled for diseases and years).

Supplementary Table S3. Global use of vector control insecticides, presented by intervention types and year (pooled for diseases).

Supplementary Table S4. Vector control insecticide use by insecticide class, presented by region, year and insecticide class (pooled for diseases).

Supplementary Table S5. Vector control insecticide use, excluding use in ITNs, by disease.

Supplementary Table S6. Pyrethroid resistance versus pyrethroid use per country.

Supplementary Table S7. Degree of insecticide resistance management for disease vector control.

## Supplementary Methods

**Estimation of spray coverage.** Data on recommended application rates were obtained from WHO[1,2], and were supplemented where needed with current manufacturers' data, as follows (in g/m$^2$): alpha-cypermethrin: 0.025, bifenthrin: 0.0375, cyfluthrin: 0.035, cypermethrin: 0.5, deltamethrin: 0.0225, etofenprox: 0.2, lambda-cyhalothrin: 0.025, permethrin: 0.5 (pyrethroids); DDT: 1.5 (organochlorine); bendiocarb: 0.4, propoxur: 1.5 (carbamates); fenitrothion: 2; malathion: 2, pirimiphos-methyl: 1 (organophosphates); and clothianidin: 0.3 (neonicotinoid). For other, less commonly used active ingredients, the application rates were projected by the average of available data for active ingredients within each insecticide class. Most larvicide compounds are unique active ingredients not recommended or used for vector adulticiding. An exception is pirimiphos-methyl that has been recommended for use in both IRS (at 1 g/m$^2$) and larviciding (at 275 g/ha); the ratio of the recommended application rates for IRS versus larviciding was used to convert the spray utility of other larvicides as required for calculation of the standard spray coverage as follows (in g/m$^2$): temephos: 0.31; fenthion: 0.24; chlorpyriphos: 0.065 (organophosphates); diflubenzuron: 0.23; methoprene: 0.11; pyriproxyfen: 0.027 (insect growth regulators); *Bacillus sphaericus*: 1.35; *Bacillus thuringiensis israelensis*: 0.76 (bacterial larvicides); and spinosad: 0.11 (spinosyn).

**Assessment of insecticide resistance management.** To study the degree of reactive resistance management, two data types were paired per country per year: publicly available data on insecticide susceptibility, and insecticide use data. For the first data type, country-specific data on insecticide susceptibility of *Anopheles* spp. determined in WHO tube tests with discriminating concentrations of insecticides were obtained from WHO's database[3]. It was assumed that anophelines tested in each country were malaria vectors, and that samples had been taken from representative sites. The mean percent mortality in bioassay results was calculated per country per year. For the second data type, the presence/absence of data on insecticide use (not limited to pyrethroids) for IRS in malaria control was determined per country per year. Country-year combinations eligible for inclusion in the analysis were those with, both, bioassay data and insecticide use data presented (data pairs). Subsequently, the bioassay data were categorized as 'confirmed resistance' in country *x* and year *y*, with <90% mortality in mosquitoes, versus 'no confirmed resistance', with ≥90% mortality including suspected resistance and full susceptibility[4]. Insecticide use data were categorized as 'pyrethroids in use' (i.e. pyrethroids used for IRS in malaria control in country *x* and year *y*) versus 'no pyrethroids in use'. To enhance temporal representation of countries over the study period, countries were eligible for inclusion only if they had at least one data pair in the period 2010–2014 and one data pair in the period 2015–2019.

**Supplementary Notes**

The following persons (anonymous when name not available) are gratefully acknowledged for their assistance in the collection and validation of data at country level, as presented alphabetically by country:

Abdul Ali Ahmadi (Afghanistan); Ranjith de Alvis (Angola); Maria Virginia Introini (Argentina); Lusine Paronyan (Armenia); A.C. Thompson (Bahamas); Raja Al Salloom (Bahrain); Shahnila Ferdousi and Sabera Sultana (Bangladesh); Kim Bautista (Belize); Anonymous (Benin); Tenzin Wangdi and Sonam Wangdi (Bhutan); Jose Laura, Favio Rodriguez, José Luis Lara, Carla Romero and Enzo Gamarra (Bolivia, Plurinational State of); Anonymous (Bosnia and Herzegovina); Gorata Boithoko and Godira Segoea (Botswana); Joyce Pereira, Joao de Souza Teixeira and Isaias Teixeira Neto (Brazil); Yacouba Savadogo (Burkina Faso); Gilbert Ntampuhwe (Burundi); Siv Sovannaroth (Cambodia); Demba Kodindo Israël (Chad); Barbara Hott Harvey (Chile); Qiyong Lui (China); Jose Fernando Valderrama Vergara, Tania Tibaduiza and Ivan Cárdenas (Colombia); Anonymous (Côte d'Ivoire); Rodrigo Marín Rodriguez and José Manuel Gutiérrez Alvardo (Costa Rica); Evelyn Martínez Cruz and José Angel Portal Miranda (Cuba); Marios Violaris (Cyprus); Mohamed Abdoulkader Guelleh (Djibouti); Angel Solis (Dominican Republic); Juliana Solórzano (Ecuador); Rolando Masis López (El Salvador); Selam Mihreteab (Eritrea); Zulisile Zulu (Eswatini); Dawit Teshome Akako and Habtamu Berhanu (Ethiopia); Litia Vatuvoka (Fiji); Anonymous (the Islamic Republic of the Gambia); Anonymous (Ghana); Kennie James (Grenada); Aida Véliz Sierra and Pedro Marcelino Yax (Guatemala); Govindra Punu and Mohini Sooklall (Guyana); Jesula Leveille (Haiti); Wilberto Montalvan (Honduras); Kalpana Baruah and Roop Kumari (India); Achmad Azhari Naufal (Indonesia); Fatemeh Nikpour (Islamic Republic of Iran); Hameeda M. Hassan (Iraq); Khalil Kanani and Nizar Maswadi (Jordan); Zhanna Shapiyeva (Kazakhstan); Kim Yun Chol (Democratic People' Republic of Korea); Samia Salem Al Dabeekh (Kuwait); Santi Maithaviphet (Lao People's Democratic Republic); Najib Aachi and Bassel El Bazzal (Lebanon); Nambinisoa Andriamananjara, Kiti Eugene and Jean Rakotoson (Madagascar); Moosa Haneef and Aishath Thimna Latheef (Maldives); Anonymous (Mali); Prem Boolaky (Mauritius); Ruy López Ridaura (Mexico); Anonymous (Republic of Moldova); Nebojsa Sekulic (Montenegro); Herrak Tachfine and Btissam Ameur (Morroco); Baltazar Candrinho (Mozambique); Thet Oo, Badri Thapa and Md. Rahman (Myanmar); Hans Angula and Stark Katokele (Namibia); Basu Pandey and Usha Kiran (Nepal); Hadiza Soumaila (Niger); Petrus Inyama and Okeke Joseph (Nigeria); Nikolina Sokolovska (North Macedonia); Khalifa Salim Alghusaini and Osama Mahmoud (Oman); Muhammad Mukhtar (Pakistan); Carlos Ruiz, Fernando Vizcaino and José Lasso (Panama); Monica Ozorio and Viviana de Egea (Paraguay); Elena Ogusuku (Peru); Emmanuel Hakizimana (Rwanda); Mohamed Alzahrani and Mohammed Al Helal (Saudi Arabia); Mamoudou Wade and Abdoulaye Diop (Senegal); Hugo Bugoro (Solomon Islands); Said Dahir Ali, Jeylani Busuri Mayow, Hassan Mohamed Jimale and Abdihafidh Yassin (Somalia); Eunice Misiani (South Africa); M Janaki, Navaratnasingam Janakan, Preshila Samaraweera and Mizaya Cader (Sri Lanka); Hmooda Kafy, Almustafa Abubaker and Mohamed Elmonshawi (Sudan); Ayman Alkhatib and Atef Al Tawil (Syrian Arab Republic); Charles Mwalimu (United Republic of Tanzania); Pongsakorn Sadakorn and Gopinath Deyer (Thailand); Octavio and Debashish Kundu (Timor Leste); Tameklou Agbeko (Togo); Naresh Nandram (Trinidad and Tobago); Jabeur Daaboub and Lamia Somai (Tunisia); Anonymous (Uganda); Shamsideen Kolawole (United Arab Emirates); Gabriela Willat (Uruguay); Tran Quang Phuc (Viet Nam); Adel Al-Jassari, Mohammed Esmail and Yassar Baheshm (Yemen); Rueben Zulu (Zambia); Wilson Chauke and Shadreck Chande (Zimbabwe).

**Table S1.** Targeted countries, with population size and annual responses, and calculation of annual weighting factor.

| Region[a] | Targeted country | Population (mln)[b] | Responding countries by year | | | | | | | | | |
|---|---|---|---|---|---|---|---|---|---|---|---|---|
| | | | 2010 | 2011 | 2012 | 2013 | 2014 | 2015 | 2016 | 2017 | 2018 | 2019 |
| **A. Population and survey responses by country** | | | | | | | | | | | | |
| AF | Algeria | 43.1 | | | | | | | | | | |
| AF | Angola | 31.8 | | | 1 | 1 | 1 | | | | | |
| AF | Benin | 11.8 | 1 | 1 | 1 | 1 | 1 | 1 | 1 | 1 | 1 | |
| AF | Botswana | 2.3 | 1 | 1 | 1 | 1 | 1 | 1 | 1 | 1 | 1 | 1 |
| AF | Burkina Faso | 20.3 | 1 | 1 | 1 | 1 | 1 | | | | 1 | 1 |
| AF | Burundi | 11.5 | | 1 | 1 | | | | | | | |
| AF | Cabo Verde | 0.5 | | | | | | | | | | |
| AF | Cameroon | 25.9 | | | | | | | | | | |
| AF | Central African Republic | 4.7 | | | | | | | | | | |
| AF | Chad | 15.9 | | | | 1 | 1 | | | | | |
| AF | Comoros | 0.9 | | | | | | | | | | |
| AF | Congo | 5.4 | | | | | | | | | | |
| AF | Democratic Rep. of Congo | 86.8 | | | | | | | | | | |
| AF | Côte d'Ivoire | 25.7 | 1 | 1 | 1 | 1 | 1 | 1 | 1 | 1 | 1 | 1 |
| AF | Djibouti | 1.0 | 1 | 1 | 1 | 1 | 1 | 1 | 1 | 1 | 1 | 1 |
| AF | Egypt | 100.4 | | | | | | | | | | |
| AF | Equatorial Guinea | 1.4 | | | | | | | | | | |
| AF | Eritrea | 3.5 | 1 | 1 | 1 | 1 | 1 | 1 | 1 | 1 | 1 | 1 |
| AF | Eswatini | 1.1 | 1 | 1 | 1 | 1 | 1 | 1 | 1 | 1 | 1 | 1 |
| AF | Ethiopia | 112.1 | 1 | 1 | 1 | 1 | 1 | 1 | 1 | 1 | 1 | 1 |
| AF | Gabon | 2.2 | | | | | | | | | | |
| AF | Islamic Rep. of the Gambia | 2.3 | 1 | 1 | 1 | 1 | 1 | | | | | |
| AF | Ghana | 30.4 | 1 | 1 | 1 | 1 | 1 | 1 | 1 | 1 | 1 | 1 |
| AF | Guinea | 12.8 | | | | | | | | | | |
| AF | Guinea-Bissau | 1.9 | | | | | | | | | | |
| AF | Kenya | 52.6 | | | | | | | | 1 | 1 | |
| AF | Lesotho | 2.1 | | | | | | | | | | |
| AF | Liberia | 4.9 | | | | | | | | | | |
| AF | Libya | 6.8 | | | | | | | | | | |
| AF | Madagascar | 27.0 | 1 | 1 | 1 | 1 | 1 | 1 | 1 | 1 | 1 | |
| AF | Malawi | 18.6 | | | | | | | | | | |
| AF | Mali | 19.7 | 1 | 1 | 1 | 1 | 1 | 1 | 1 | 1 | 1 | 1 |
| AF | Mauritania | 4.5 | | | | | | | | | | |
| AF | Mauritius | 1.3 | 1 | 1 | 1 | 1 | 1 | | | | | |
| AF | Morocco | 36.5 | 1 | 1 | 1 | 1 | 1 | 1 | 1 | 1 | 1 | 1 |
| AF | Mozambique | 30.4 | 1 | 1 | 1 | 1 | 1 | 1 | 1 | 1 | 1 | 1 |
| AF | Namibia | 2.5 | 1 | 1 | 1 | 1 | 1 | 1 | 1 | 1 | 1 | 1 |
| AF | Niger | 23.3 | 1 | | | 1 | 1 | | | | | |
| AF | Nigeria | 201.0 | 1 | 1 | | 1 | | | | | | |
| AF | Rwanda | 12.6 | 1 | 1 | 1 | 1 | 1 | 1 | 1 | 1 | 1 | 1 |

| | | | | | | | | | | | | |
|---|---|---|---|---|---|---|---|---|---|---|---|---|
| AF | Sao Tomé & Principe | 0.2 | | | | | | | | | | |
| AF | Senegal | 16.3 | 1 | 1 | 1 | 1 | 1 | 1 | 1 | 1 | | |
| AF | Seychelles | 0.1 | | | | | | | | | | |
| AF | Sierra Leone | 7.8 | | | | | | | | | | |
| AF | Somalia | 15.4 | | | | 1 | 1 | | | | | |
| AF | South Africa | 58.6 | 1 | 1 | 1 | 1 | 1 | 1 | 1 | 1 | 1 | 1 |
| AF | South Sudan | 11.1 | | | | | | | | | | |
| AF | Sudan | 42.8 | 1 | 1 | 1 | 1 | 1 | 1 | 1 | 1 | | |
| AF | United Rep. of Tanzania | 58.0 | 1 | 1 | 1 | 1 | 1 | 1 | 1 | 1 | 1 | |
| AF | Togo | 8.1 | 1 | | | | | | | | | |
| AF | Tunisia | 11.7 | 1 | 1 | 1 | 1 | 1 | | | | | |
| AF | Uganda | 44.3 | 1 | 1 | 1 | 1 | 1 | 1 | 1 | 1 | 1 | 1 |
| AF | Zambia | 17.9 | 1 | 1 | 1 | 1 | 1 | 1 | 1 | 1 | 1 | 1 |
| AF | Zimbabwe | 14.6 | 1 | 1 | 1 | 1 | 1 | 1 | 1 | 1 | 1 | 1 |
| AP | Afghanistan | 38.0 | | | 1 | | 1 | 1 | | | 1 | 1 |
| AP | Bahrain | 1.6 | | 1 | 1 | 1 | 1 | 1 | 1 | 1 | 1 | 1 |
| AP | Bangladesh | 163.0 | 1 | 1 | 1 | 1 | 1 | 1 | 1 | 1 | 1 | 1 |
| AP | Bhutan | 0.8 | 1 | | | | 1 | 1 | 1 | | 1 | 1 |
| AP | Brunei Darussalam | 0.4 | | | | | | | | | | |
| AP | Cambodia | 16.5 | 1 | 1 | 1 | 1 | 1 | 1 | 1 | 1 | 1 | 1 |
| AP | China | 1433.8 | 1 | 1 | 1 | 1 | 1 | 1 | 1 | 1 | 1 | 1 |
| AP | Cook Islands | 0.0 | | | | | | | | | | |
| AP | Cyprus | 1.2 | 1 | 1 | 1 | 1 | 1 | | | | | |
| AP | Fiji | 0.9 | 1 | 1 | 1 | 1 | 1 | 1 | 1 | 1 | 1 | 1 |
| AP | India | 1366.4 | 1 | 1 | 1 | 1 | 1 | 1 | 1 | 1 | 1 | 1 |
| AP | Indonesia | 270.6 | 1 | 1 | 1 | 1 | 1 | 1 | 1 | 1 | 1 | 1 |
| AP | Islamic Rep. of Iran | 82.9 | 1 | 1 | 1 | 1 | 1 | 1 | 1 | 1 | | |
| AP | Iraq | 39.3 | 1 | 1 | 1 | 1 | 1 | 1 | 1 | 1 | 1 | 1 |
| AP | Jordan | 10.1 | 1 | 1 | 1 | 1 | 1 | 1 | 1 | 1 | 1 | 1 |
| AP | Kazakhstan | 18.6 | 1 | 1 | 1 | 1 | 1 | | | | | |
| AP | Kiribati | 0.1 | | | | | | | | | | |
| AP | D.P.R. Korea | 25.7 | 1 | 1 | 1 | 1 | 1 | 1 | 1 | 1 | | |
| AP | Kuwait | 4.2 | 1 | 1 | 1 | 1 | 1 | 1 | 1 | 1 | | |
| AP | Kyrgyzstan | 6.4 | | | | | | | | | | |
| AP | Lao P.D.R. | 7.2 | 1 | 1 | 1 | 1 | 1 | 1 | 1 | 1 | 1 | 1 |
| AP | Lebanon | 6.9 | 1 | 1 | 1 | 1 | 1 | 1 | 1 | 1 | 1 | 1 |
| AP | Malaysia | 31.9 | | | | | | | | | | |
| AP | Maldives | 0.5 | 1 | 1 | 1 | 1 | 1 | 1 | | | | |
| AP | Marshall Islands | 0.1 | | | | | | | | | | |
| AP | Fed. States of Micronesia | 0.1 | | | | | | | | | | |
| AP | Mongolia | 3.2 | | | | | | | | | | |
| AP | Myanmar | 54.0 | 1 | 1 | 1 | 1 | 1 | 1 | 1 | 1 | 1 | 1 |
| AP | Nauru | 0.0 | | | | | | | | | | |
| AP | Nepal | 28.6 | 1 | 1 | 1 | 1 | 1 | 1 | 1 | 1 | 1 | 1 |
| AP | Niue | 0.0 | | | | | | | | | | |
| AP | Oman | 5.0 | 1 | 1 | 1 | 1 | 1 | | | | | |
| AP | Pakistan | 216.6 | 1 | 1 | 1 | 1 | 1 | 1 | 1 | 1 | 1 | 1 |
| AP | Palau | 0.0 | | | | | | | | | | |

| | | | | | | | | | | | | |
|---|---|---|---|---|---|---|---|---|---|---|---|---|
| AP | Papua New Guinea | 8.8 | | | | | | | | | | |
| AP | Philippines | 108.1 | | | | | | | | | | |
| AP | Qatar | 2.8 | | | | | | | | | | |
| AP | Samoa | 0.2 | | | | | | | | | | |
| AP | Saudi Arabia | 34.3 | 1 | 1 | 1 | 1 | 1 | 1 | 1 | 1 | 1 | 1 |
| AP | Singapore | 5.8 | | | | | | | | | | |
| AP | Solomon Islands | 0.7 | 1 | 1 | 1 | 1 | 1 | 1 | 1 | 1 | 1 | 1 |
| AP | Sri Lanka | 21.3 | 1 | 1 | 1 | 1 | 1 | 1 | 1 | 1 | 1 | 1 |
| AP | Syrian Arab Rep. | 17.1 | 1 | 1 | 1 | 1 | 1 | 1 | 1 | 1 | 1 | 1 |
| AP | Tajikistan | 9.3 | | | | | | | | | | |
| AP | Thailand | 69.6 | 1 | 1 | 1 | 1 | 1 | 1 | | | | |
| AP | Timor-Léste | 1.3 | | | | | | 1 | 1 | 1 | 1 | 1 |
| AP | Tonga | 0.1 | | | | | | | | | | |
| AP | Tuvalu | 0.0 | | | | | | | | | | |
| AP | United Arab Emirates | 9.8 | 1 | 1 | 1 | 1 | 1 | 1 | 1 | 1 | 1 | 1 |
| AP | Uzbekistan | 33.0 | | | | | | | | | | |
| AP | Vanuatu | 0.3 | | | | | | | | | | |
| AP | Viet Nam | 96.5 | 1 | 1 | 1 | 1 | 1 | 1 | 1 | 1 | 1 | 1 |
| AP | Yemen | 29.2 | 1 | 1 | 1 | 1 | 1 | 1 | 1 | 1 | 1 | 1 |
| LA | Antigua and Barbuda | 0.1 | | | | | | | | | | |
| LA | Argentina | 44.8 | 1 | 1 | 1 | 1 | 1 | 1 | | | | |
| LA | Bahamas | 0.4 | 1 | 1 | 1 | 1 | 1 | 1 | 1 | 1 | | |
| LA | Barbados | 0.3 | | | | | | | | | | |
| LA | Belize | 0.4 | 1 | 1 | 1 | 1 | 1 | 1 | 1 | 1 | 1 | 1 |
| LA | Bolivia (Plurinational St. of) | 11.5 | 1 | 1 | 1 | 1 | 1 | 1 | 1 | 1 | 1 | 1 |
| LA | Brazil | 211.0 | 1 | 1 | 1 | 1 | 1 | 1 | 1 | 1 | 1 | 1 |
| LA | Chile | 19.0 | 1 | 1 | 1 | 1 | 1 | 1 | 1 | 1 | 1 | 1 |
| LA | Colombia | 50.3 | 1 | 1 | 1 | | 1 | 1 | 1 | 1 | 1 | 1 |
| LA | Costa Rica | 5.0 | 1 | 1 | 1 | 1 | 1 | 1 | 1 | 1 | 1 | 1 |
| LA | Cuba | 11.3 | 1 | 1 | 1 | 1 | 1 | 1 | 1 | 1 | 1 | 1 |
| LA | Dominica | 0.1 | | | | | | | | | | |
| LA | Dominican Republic (the) | 10.7 | 1 | 1 | 1 | 1 | 1 | 1 | 1 | 1 | | |
| LA | Ecuador | 17.4 | 1 | 1 | 1 | 1 | 1 | 1 | 1 | 1 | | |
| LA | El Salvador | 6.5 | 1 | 1 | 1 | 1 | 1 | 1 | 1 | 1 | | |
| LA | Grenada | 0.1 | 1 | 1 | 1 | 1 | 1 | 1 | 1 | 1 | | |
| LA | Guatemala | 17.6 | 1 | 1 | 1 | 1 | 1 | 1 | 1 | 1 | 1 | 1 |
| LA | Guyana | 0.8 | | | | | 1 | 1 | | | | |
| LA | Haiti | 11.3 | | | | 1 | | 1 | 1 | 1 | 1 | 1 |
| LA | Honduras | 9.7 | | | | | | | | | | |
| LA | Jamaica | 2.9 | | | | | | | | | | |
| LA | Mexico | 127.6 | 1 | 1 | 1 | 1 | 1 | 1 | 1 | 1 | 1 | 1 |
| LA | Nicaragua | 6.5 | | | | | | | | | | |
| LA | Panama | 4.2 | 1 | 1 | 1 | 1 | 1 | 1 | | | | |
| LA | Paraguay | 7.0 | 1 | 1 | 1 | 1 | 1 | 1 | 1 | 1 | 1 | 1 |
| LA | Peru | 32.5 | 1 | 1 | 1 | | 1 | 1 | 1 | 1 | | |
| LA | Saint Kitts & Nevis | 0.1 | | | | | | | | | | |
| LA | Saint Lucia | 0.2 | | | | | | | | | | |
| LA | Saint Vincent & Grenadines | 0.1 | | | | | | | | | | |

| | | | | | | | | | | | | |
|---|---|---|---|---|---|---|---|---|---|---|---|---|
| LA | Suriname | 0.6 | | | | | | | | | | |
| LA | Trinidad & Tobago | 1.4 | 1 | 1 | 1 | 1 | 1 | 1 | | | | |
| LA | Uruguay | 3.5 | 1 | 1 | 1 | 1 | 1 | 1 | 1 | 1 | | |
| LA | Venezuela (Bolivarian Rep. of) | 28.5 | | | | | | | | | | |
| **Excluded from analysis:** | | | | | | | | | | | | |
| EE | Albania | 2.9 | | | | | | | | | | |
| EE | Armenia | 3.0 | 1 | 1 | 1 | 1 | 1 | | | | | |
| EE | Azerbaijan | 10.0 | | | | | | | | | | |
| EE | Bosnia & Herzegovina | 3.3 | 1 | 1 | 1 | 1 | 1 | | | | | |
| EE | Bulgaria | 7.0 | | | | | | | | | | |
| EE | Croatia | 4.1 | | | | | | | | | | |
| EE | Estonia | 1.3 | | | | | | | | | | |
| EE | Georgia | 4.0 | | | | | | | | | | |
| EE | Hungary | 9.7 | | | | | | | | | | |
| EE | Republic of Moldova | 4.0 | | | 1 | 1 | 1 | | | | | |
| EE | Montenegro | 0.6 | 1 | 1 | 1 | 1 | 1 | | | | | |
| EE | North Macedonia | 2.1 | 1 | 1 | 1 | 1 | 1 | | | | | |
| EE | Romania | 19.4 | | | | | | | | | | |
| EE | Russian Federation | 145.9 | | | | | | | | | | |
| EE | Serbia | 8.8 | | | | | | | | | | |
| EE | Ukraine | 44.0 | | | | | | | | | | |
| WE | France | 65.1 | | | | | | | | | | |
| WE | Greece | 10.5 | | | | | | | | | | |
| WE | Israel | 8.5 | | | | | | | | | | |
| WE | Italy | 60.6 | | | | | | | | | | |
| WE | Portugal | 10.2 | | | | | | | | | | |
| WE | Spain | 46.7 | | | | | | | | | | |
| WE | Switzerland | 8.6 | | | | | | | | | | |
| WE | Turkey | 83.4 | | | | | | | | | | |
| **B. Population and survey responses by region** | | | | | | | | | | | | |
| AF | | 1306.3 | 28 | 27 | 27 | 30 | 29 | 21 | 21 | 22 | 21 | 17 |
| AP | | 4282.8 | 29 | 29 | 30 | 29 | 31 | 29 | 26 | 25 | 24 | 24 |
| LA | | 643.5 | 20 | 20 | 20 | 19 | 21 | 22 | 18 | 18 | 11 | 11 |
| EE (excluded from analysis) | | 267.2 | 4 | 4 | 5 | 5 | 5 | - | - | - | - | - |
| WE (excluded from analysis) | | 293.7 | - | - | - | - | - | - | - | - | - | - |
| All | | 6793.5 | 81 | 80 | 82 | 83 | 86 | 72 | 65 | 65 | 56 | 52 |
| All except EE and WE | | 6232.6 | 77 | 76 | 77 | 78 | 81 | 72 | 65 | 65 | 56 | 52 |
| **C. Calculation of annual weighting factor** | | | | | | | | | | | | |
| Population of responding countries[c] | | | 5450 | 5431 | 5300 | 5435 | 5345 | 5211 | 5051 | 5103 | 4919 | 4770 |
| Population of targeted countries[c] | | | 6233 | 6233 | 6233 | 6233 | 6233 | 6233 | 6233 | 6233 | 6233 | 6233 |
| Annual weighting factor | | | 0.874 | 0.871 | 0.850 | 0.872 | 0.858 | 0.836 | 0.810 | 0.819 | 0.789 | 0.765 |

[a]United Nations Regional Groups: AF, African; AP, Asia-Pacific; LA, Latin American & Caribbean; EE, Eastern European; WE, Western European & Others [5]

[b]World Population Prospects 2019, Dept Economic and Social Affairs, Population Division, United Nations, New York [6]

[c]All regions except EE and WE

**Table S2.** Use of active ingredients for vector control spraying operations in ranking order (pooled for diseases and years).*

| Rank | Insecticide compound | Insecticide class | Region | | | |
|---|---|---|---|---|---|---|
| | | | African | Asia-Pacific | Latin American & Caribbean | All |
| 1 | DDT | organochlorine | 206.27 | 1822.34 | 0.00 | 2028.61 |
| 2 | Deltamethrin | pyrethroid | 719.44 | 615.81 | 545.09 | 1880.35 |
| 3 | Lambdacyhalothrin | pyrethroid | 580.59 | 414.94 | 108.66 | 1104.19 |
| 4 | Alphacypermethrin | pyrethroid | 35.44 | 770.10 | 446.79 | 1252.33 |
| 5 | Bendiocarb | carbamate | 437.17 | 31.95 | 244.87 | 713.99 |
| 6 | Malathion | organophosphate | 355.43 | 1.22 | 15.75 | 372.40 |
| 7 | Pirimiphos-methyl | organophosphate | 0.08 | 33.60 | 397.70 | 431.38 |
| 8 | Pyriproxifen | insect growth regulator | 24.89 | 89.04 | 163.70 | 277.63 |
| 9 | Temephos | organophosphate | 0.00 | 41.13 | 270.40 | 311.53 |
| 10 | Propoxur | carbamate | 103.03 | 3.52 | 110.48 | 217.03 |
| 11 | Spinosad | spynosyn | 105.57 | 3.50 | 0.00 | 109.07 |
| 12 | Clothianidin | neonicotinoid | 0.00 | 46.11 | 86.71 | 132.81 |
| 13 | *Bacillus thuringiensis israelensis* | bacterial larvicide | 0.14 | 48.29 | 49.83 | 98.26 |
| 14 | Chlorpyrifos | organophosphate | 0.00 | 3.07 | 81.29 | 84.35 |
| 15 | Bifenthrin | pyrethroid | 7.94 | 11.81 | 28.99 | 48.75 |
| 16 | Permethrin | pyrethroid | 0.00 | 8.78 | 47.55 | 56.33 |
| 17 | Cypermethrin | pyrethroid | 0.00 | 34.45 | 0.00 | 34.45 |
| 18 | Diflubenzeron | insect growth regulator | 0.12 | 14.43 | 32.86 | 47.41 |
| 19 | Beta-cypermethrin | pyrethroid | 0.39 | 19.47 | 20.30 | 40.17 |
| 20 | Cyfluthrin | pyrethroid | 0.32 | 27.02 | 0.62 | 27.96 |
| 21 | Etofenprox | pyrethroid | 1.40 | 7.37 | 19.40 | 28.17 |
| 22 | Cyphenothrin | pyrethroid | 0.00 | 14.89 | 0.00 | 14.89 |
| 23 | Fenthion | organophosphate | 0.00 | 9.29 | 0.26 | 9.68 |
| 24 | Beta-cyfluthrin | pyrethroid | 0.00 | 7.10 | 0.00 | 7.10 |
| 25 | Phoxim | organophosphate | 0.00 | 6.80 | 0.00 | 6.80 |
| 26 | Phenothrin | pyrethroid | 0.00 | 0.00 | 5.60 | 5.60 |
| 27 | Fenitrothion | organophosphate | 0.00 | 2.30 | 0.00 | 2.30 |
| 28 | Novaluron | insect growth regulator | 0.00 | 0.00 | 4.39 | 4.39 |
| 29 | Dichlorvos | organophosphate | 0.00 | 0.00 | 3.51 | 3.51 |
| 30 | Pyrethrum | pyrethroid | 0.00 | 0.00 | 3.49 | 3.49 |
| 31 | *Bacillus sphaericus* | bacterial larvicide | 0.00 | 1.52 | 0.00 | 1.52 |
| 32 | d-tetramethrin+cyphenothrin | pyrethroid | 0.00 | 1.26 | 0.00 | 1.26 |
| 33 | Tetramethrin | pyrethroid | 0.00 | 0.89 | 0.00 | 0.89 |
| 34 | Transfluthrin | pyrethroid | 0.00 | 0.25 | 1.18 | 1.43 |
| 35 | Permethrin-allethrin | pyrethroid | 0.00 | 0.53 | 0.19 | 0.73 |
| 36 | Imidacloprid | neonicotinoid | 0.00 | 0.00 | 1.12 | 1.12 |
| 37 | Methoprene | insect growth regulator | 0.00 | 0.00 | 0.93 | 0.93 |

| | | | | | | |
|---|---|---|---|---|---|---|
| 38 | S-bioallethrin | pyrethroid | 0.00 | 0.19 | 0.00 | 0.19 |
| 39 | Pyrethrin | pyrethroid | 0.00 | 0.13 | 0.00 | 0.13 |
| 40 | Deltamethrin+S-bioallethrin+Piperonyl butoxide | pyrethroid | 0.00 | 0.11 | 0.00 | 0.11 |
| 41 | Chlorpyrifos-ethyl | organophosphate | 0.10 | 0.00 | 0.00 | 0.10 |
| 42 | Zeta-cypermethrin | pyrethroid | 0.00 | 0.00 | 0.18 | 0.18 |
| 43 | Diazinon | organophosphate | 0.00 | 0.04 | 0.00 | 0.04 |
| 44 | Cyfluthrin+Tetramethrin | pyrethroid | 0.00 | 0.01 | 0.00 | 0.01 |

*Results expressed in standard spray coverage (per million $m^2$)

**Table S3.** Global use of vector control insecticides, presented by intervention types and year (pooled for diseases).*

| Year | Intervention method | | | | |
|---|---|---|---|---|---|
| | ITN | ITN-kit | Residual spraying | Space spraying | Larviciding |
| 2010 | 7804.26 | 419.30 | 8328.99 | 641.11 | 605.16 |
| 2011 | 6047.71 | 599.33 | 12060.84 | 843.15 | 529.92 |
| 2012 | 5276.79 | 436.53 | 8421.97 | 619.34 | 728.93 |
| 2013 | 10681.60 | 57.77 | 8077.07 | 1212.33 | 493.79 |
| 2014 | 13331.09 | 39.68 | 8733.75 | 1477.64 | 1296.49 |
| 2015 | 13033.82 | 32.24 | 6885.17 | 1506.39 | 1336.16 |
| 2016 | 9864.13 | 133.80 | 5969.08 | 1399.26 | 1290.33 |
| 2017 | 17243.07 | 35.72 | 5331.47 | 996.14 | 801.59 |
| 2018 | 14424.59 | 32.48 | 5605.18 | 701.73 | 842.05 |
| 2019 | 19148.49 | 122.58 | 3860.40 | 2184.33 | 854.92 |

*Results expressed in standard spray coverage ($10^6$ $m^2$)

**Table S4.** Vector control insecticide use by insecticide class, presented by region, year, and insecticide class (pooled for diseases).

| Region | Year | Insecticide class* | | | | | | |
|---|---|---|---|---|---|---|---|---|
| | | Pyrethroids (ITN) | Pyrethroids (other) | Organo-chlorines | Organo-phosphates | Carbamates | Neonico-tinoids | Other classes** |
| African | 2010 | 6766.30 | 2472.76 | 240.50 | 42.88 | 211.47 | 0.00 | 3.87 |
| | 2011 | 4398.57 | 2245.66 | 266.67 | 36.62 | 436.48 | 0.00 | 0.04 |
| | 2012 | 4031.54 | 1935.98 | 146.16 | 56.16 | 1125.75 | 0.00 | 0.34 |
| | 2013 | 9404.84 | 1592.60 | 182.36 | 87.72 | 1317.98 | 0.00 | 0.56 |
| | 2014 | 11726.99 | 1325.45 | 163.37 | 250.55 | 897.58 | 0.00 | 0.01 |
| | 2015 | 11389.07 | 1226.08 | 162.81 | 337.46 | 1099.08 | 0.00 | 0.01 |
| | 2016 | 8374.29 | 1040.99 | 235.08 | 731.58 | 182.52 | 0.00 | 0.15 |
| | 2017 | 13875.12 | 731.18 | 250.38 | 1048.46 | 131.04 | 0.00 | 0.27 |
| | 2018 | 11988.95 | 353.50 | 242.19 | 847.45 | 0.08 | 387.87 | 0.00 |
| | 2019 | 14910.60 | 528.41 | 173.14 | 366.09 | 0.00 | 667.81 | 38.08 |
| Asia-Pacific and Latin American & Caribbean | 2010 | 1457.26 | 2997.60 | 2240.05 | 610.14 | 522.63 | 0.00 | 233.37 |
| | 2011 | 2248.47 | 7018.12 | 2456.11 | 545.51 | 281.14 | 0.00 | 147.58 |
| | 2012 | 1681.77 | 2919.61 | 2423.18 | 710.15 | 164.81 | 0.00 | 288.11 |
| | 2013 | 1334.53 | 3117.72 | 2363.61 | 829.60 | 133.83 | 0.00 | 157.19 |
| | 2014 | 1643.77 | 3017.53 | 2199.23 | 838.04 | 1799.55 | 0.00 | 1016.58 |
| | 2015 | 1676.98 | 2774.03 | 1683.77 | 960.32 | 326.31 | 0.00 | 1157.86 |
| | 2016 | 1623.63 | 2565.81 | 1562.93 | 1048.42 | 211.48 | 0.00 | 1079.72 |
| | 2017 | 3403.66 | 2240.71 | 1213.63 | 723.01 | 216.97 | 0.01 | 573.55 |
| | 2018 | 2468.12 | 2589.47 | 1228.99 | 732.72 | 112.90 | 0.35 | 653.36 |
| | 2019 | 4331.32 | 2458.16 | 851.95 | 1099.75 | 138.57 | 43.94 | 571.82 |

*Results expressed in standard spray coverage ($10^6$ $m^2$)

**Bacterial larvicides, insect growth regulators and spinosyns

**Table S5.** Vector control insecticide use, excluding use in ITNs, by disease.

| | | | Insecticide class* | | | | | |
|---|---|---|---|---|---|---|---|---|
| Disease | Region | Year | Pyrethroids | Organo-chlorines | Organo-phosphates | Carbamates | Neonico-tinoids | Other classes** |
| Malaria | African | 2010 | 2430.50 | 240.50 | 42.29 | 211.47 | 0.00 | 3.14 |
| | | 2011 | 2212.76 | 266.67 | 36.17 | 421.18 | 0.00 | 0.03 |
| | | 2012 | 1933.46 | 146.16 | 55.82 | 1099.47 | 0.00 | 0.34 |
| | | 2013 | 1576.08 | 182.36 | 87.37 | 1285.11 | 0.00 | 0.56 |
| | | 2014 | 1285.92 | 163.37 | 250.06 | 871.92 | 0.00 | 0.00 |
| | | 2015 | 1216.46 | 162.81 | 337.46 | 1045.83 | 0.00 | 0.00 |
| | | 2016 | 1031.02 | 235.08 | 731.58 | 124.47 | 0.00 | 0.15 |
| | | 2017 | 723.57 | 250.38 | 1048.46 | 70.06 | 0.00 | 0.18 |
| | | 2018 | 353.27 | 242.19 | 847.45 | 0.08 | 387.87 | 0.00 |
| | | 2019 | 526.07 | 173.14 | 365.88 | 0.00 | 667.81 | 0.00 |
| | Asia-Pacific | 2010 | 1629.94 | 1662.18 | 53.07 | 0.00 | 0.00 | 92.30 |
| | | 2011 | 1585.81 | 1564.09 | 51.31 | 0.00 | 0.00 | 30.12 |
| | | 2012 | 1363.09 | 1522.81 | 95.46 | 3.94 | 0.00 | 37.56 |
| | | 2013 | 1133.50 | 1342.95 | 54.07 | 7.65 | 0.00 | 17.81 |
| | | 2014 | 1196.59 | 1379.08 | 60.84 | 29.74 | 0.00 | 270.91 |
| | | 2015 | 1183.24 | 1635.92 | 22.31 | 48.25 | 0.00 | 248.42 |
| | | 2016 | 1383.05 | 1562.93 | 11.18 | 47.79 | 0.00 | 20.53 |
| | | 2017 | 1126.47 | 1213.63 | 7.64 | 58.18 | 0.00 | 13.56 |
| | | 2018 | 1207.42 | 1228.99 | 4.53 | 19.57 | 0.00 | 17.97 |
| | | 2019 | 1335.31 | 851.95 | 4.00 | 16.88 | 35.03 | 16.95 |
| | Latin American & Caribbean | 2010 | 353.04 | 0.00 | 106.98 | 0.11 | 0.00 | 1.12 |
| | | 2011 | 1624.23 | 0.00 | 79.46 | 0.13 | 0.00 | 1.52 |
| | | 2012 | 342.01 | 0.00 | 70.16 | 0.08 | 0.00 | 9.84 |
| | | 2013 | 298.64 | 0.00 | 71.35 | 0.25 | 0.00 | 1.44 |
| | | 2014 | 267.95 | 0.00 | 78.41 | 0.11 | 0.00 | 0.53 |
| | | 2015 | 298.50 | 0.00 | 94.19 | 29.67 | 0.00 | 0.54 |
| | | 2016 | 96.43 | 0.00 | 6.88 | 0.06 | 0.00 | 0.74 |
| | | 2017 | 120.96 | 0.00 | 8.49 | 0.16 | 0.00 | 0.61 |
| | | 2018 | 68.11 | 0.00 | 5.56 | 0.20 | 0.00 | 1.12 |
| | | 2019 | 81.00 | 0.00 | 5.37 | 0.22 | 0.00 | 1.64 |
| Dengue | African | 2010 | 6.53 | 0.00 | 0.59 | 0.00 | 0.00 | 0.73 |
| | | 2011 | 3.15 | 0.00 | 0.45 | 0.00 | 0.00 | 0.00 |
| | | 2012 | 2.52 | 0.00 | 0.34 | 0.00 | 0.00 | 0.00 |
| | | 2013 | 2.76 | 0.00 | 0.36 | 0.00 | 0.00 | 0.00 |
| | | 2014 | 7.14 | 0.00 | 0.49 | 0.00 | 0.00 | 0.00 |
| | | 2015 | 5.92 | 0.00 | 0.00 | 0.00 | 0.00 | 0.00 |
| | | 2016 | 9.97 | 0.00 | 0.00 | 0.00 | 0.00 | 0.00 |
| | | 2017 | 6.25 | 0.00 | 0.00 | 0.00 | 0.00 | 0.09 |
| | | 2018 | 0.12 | 0.00 | 0.00 | 0.00 | 0.00 | 0.07 |
| | | 2019 | 2.32 | 0.00 | 0.21 | 0.00 | 0.00 | 0.00 |

| | | | | | | | | |
|---|---|---|---|---|---|---|---|---|
| | Asia-Pacific | 2010 | 31.73 | 0.00 | 48.92 | 0.00 | 0.00 | 89.19 |
| | | 2011 | 32.16 | 0.00 | 64.30 | 0.79 | 0.00 | 28.09 |
| | | 2012 | 111.87 | 0.00 | 51.40 | 0.31 | 0.00 | 34.28 |
| | | 2013 | 267.28 | 0.00 | 112.38 | 0.32 | 0.00 | 15.55 |
| | | 2014 | 532.73 | 0.00 | 186.11 | 1.21 | 0.00 | 268.89 |
| | | 2015 | 368.73 | 0.00 | 88.34 | 0.00 | 0.00 | 248.51 |
| | | 2016 | 311.78 | 0.00 | 83.09 | 0.12 | 0.00 | 24.04 |
| | | 2017 | 299.00 | 0.00 | 104.24 | 0.14 | 0.00 | 18.68 |
| | | 2018 | 781.49 | 0.00 | 114.59 | 23.31 | 0.00 | 27.31 |
| | | 2019 | 487.58 | 0.00 | 214.05 | 8.69 | 0.00 | 37.21 |
| | Latin American & Caribbean | 2010 | 224.81 | 0.00 | 397.88 | 267.28 | 0.00 | 50.77 |
| | | 2011 | 1569.94 | 0.00 | 350.37 | 131.23 | 0.00 | 87.84 |
| | | 2012 | 291.57 | 0.00 | 493.09 | 76.77 | 0.00 | 206.42 |
| | | 2013 | 552.69 | 0.00 | 591.75 | 86.65 | 0.00 | 122.39 |
| | | 2014 | 425.48 | 0.00 | 512.67 | 935.72 | 0.00 | 476.25 |
| | | 2015 | 413.81 | 0.00 | 755.48 | 209.74 | 0.00 | 660.38 |
| | | 2016 | 163.87 | 0.00 | 947.27 | 156.60 | 0.00 | 1034.41 |
| | | 2017 | 154.03 | 0.00 | 602.64 | 153.06 | 0.01 | 540.70 |
| | | 2018 | 46.92 | 0.00 | 608.03 | 63.27 | 0.35 | 606.97 |
| | | 2019 | 165.60 | 0.00 | 876.32 | 107.12 | 8.91 | 516.01 |
| Leishmaniasis | African | 2010 | 35.72 | 0.00 | 0.00 | 0.00 | 0.00 | 0.00 |
| | | 2011 | 29.75 | 0.00 | 0.00 | 15.30 | 0.00 | 0.00 |
| | | 2012 | 0.00 | 0.00 | 0.00 | 26.28 | 0.00 | 0.00 |
| | | 2013 | 13.76 | 0.00 | 0.00 | 32.88 | 0.00 | 0.00 |
| | | 2014 | 32.39 | 0.00 | 0.00 | 25.65 | 0.00 | 0.00 |
| | | 2015 | 3.70 | 0.00 | 0.00 | 53.25 | 0.00 | 0.00 |
| | | 2016 | 0.00 | 0.00 | 0.00 | 58.04 | 0.00 | 0.00 |
| | | 2017 | 1.36 | 0.00 | 0.00 | 60.98 | 0.00 | 0.00 |
| | | 2018 | 0.11 | 0.00 | 0.00 | 0.00 | 0.00 | 0.00 |
| | | 2019 | 0.01 | 0.00 | 0.00 | 0.00 | 0.00 | 0.00 |
| | Asia-Pacific | 2010 | 258.08 | 577.87 | 0.00 | 13.04 | 0.00 | 0.00 |
| | | 2011 | 343.65 | 892.02 | 0.06 | 14.79 | 0.00 | 0.00 |
| | | 2012 | 331.50 | 900.36 | 0.04 | 11.44 | 0.00 | 0.00 |
| | | 2013 | 416.36 | 1020.67 | 0.05 | 20.93 | 0.00 | 0.00 |
| | | 2014 | 246.61 | 820.14 | 0.00 | 7.68 | 0.00 | 0.00 |
| | | 2015 | 310.98 | 47.85 | 0.00 | 7.15 | 0.00 | 0.00 |
| | | 2016 | 319.25 | 0.00 | 0.00 | 3.17 | 0.00 | 0.00 |
| | | 2017 | 219.36 | 0.00 | 0.00 | 2.67 | 0.00 | 0.00 |
| | | 2018 | 247.16 | 0.00 | 0.00 | 2.58 | 0.00 | 0.00 |
| | | 2019 | 269.33 | 0.00 | 0.00 | 4.29 | 0.00 | 0.00 |
| | Latin American & Caribbean | 2010 | 229.53 | 0.00 | 1.65 | 0.00 | 0.00 | 0.00 |
| | | 2011 | 309.48 | 0.00 | 0.00 | 0.00 | 0.00 | 0.00 |
| | | 2012 | 210.46 | 0.00 | 0.00 | 0.00 | 0.00 | 0.00 |
| | | 2013 | 193.74 | 0.00 | 0.00 | 0.00 | 0.00 | 0.00 |

| | | | | | | | | |
|---|---|---|---|---|---|---|---|---|
| | | 2014 | 129.97 | 0.00 | 0.00 | 0.00 | 0.00 | 0.00 |
| | | 2015 | 56.89 | 0.00 | 0.00 | 0.00 | 0.00 | 0.00 |
| | | 2016 | 118.11 | 0.00 | 0.00 | 0.00 | 0.00 | 0.00 |
| | | 2017 | 129.25 | 0.00 | 0.00 | 0.00 | 0.00 | 0.00 |
| | | 2018 | 105.25 | 0.00 | 0.00 | 0.00 | 0.00 | 0.00 |
| | | 2019 | 52.22 | 0.00 | 0.00 | 0.00 | 0.00 | 0.00 |
| Chagas disease | African | 2010 | 0.00 | 0.00 | 0.00 | 0.00 | 0.00 | 0.00 |
| | | 2011 | 0.00 | 0.00 | 0.00 | 0.00 | 0.00 | 0.00 |
| | | 2012 | 0.00 | 0.00 | 0.00 | 0.00 | 0.00 | 0.00 |
| | | 2013 | 0.00 | 0.00 | 0.00 | 0.00 | 0.00 | 0.00 |
| | | 2014 | 0.00 | 0.00 | 0.00 | 0.00 | 0.00 | 0.00 |
| | | 2015 | 0.00 | 0.00 | 0.00 | 0.00 | 0.00 | 0.00 |
| | | 2016 | 0.00 | 0.00 | 0.00 | 0.00 | 0.00 | 0.00 |
| | | 2017 | 0.00 | 0.00 | 0.00 | 0.00 | 0.00 | 0.00 |
| | | 2018 | 0.00 | 0.00 | 0.00 | 0.00 | 0.00 | 0.00 |
| | | 2019 | 0.00 | 0.00 | 0.00 | 0.00 | 0.00 | 0.00 |
| | Asia-Pacific | 2010 | 0.00 | 0.00 | 0.00 | 0.00 | 0.00 | 0.00 |
| | | 2011 | 0.00 | 0.00 | 0.00 | 0.00 | 0.00 | 0.00 |
| | | 2012 | 0.00 | 0.00 | 0.00 | 0.00 | 0.00 | 0.00 |
| | | 2013 | 0.00 | 0.00 | 0.00 | 0.00 | 0.00 | 0.00 |
| | | 2014 | 0.00 | 0.00 | 0.00 | 0.00 | 0.00 | 0.00 |
| | | 2015 | 0.00 | 0.00 | 0.00 | 0.00 | 0.00 | 0.00 |
| | | 2016 | 0.00 | 0.00 | 0.00 | 0.00 | 0.00 | 0.00 |
| | | 2017 | 0.00 | 0.00 | 0.00 | 0.00 | 0.00 | 0.00 |
| | | 2018 | 0.00 | 0.00 | 0.00 | 0.00 | 0.00 | 0.00 |
| | | 2019 | 0.00 | 0.00 | 0.00 | 0.00 | 0.00 | 0.00 |
| | Latin American & Caribbean | 2010 | 270.47 | 0.00 | 1.65 | 242.20 | 0.00 | 0.00 |
| | | 2011 | 1552.85 | 0.00 | 0.00 | 134.20 | 0.00 | 0.00 |
| | | 2012 | 270.02 | 0.00 | 0.00 | 72.26 | 0.00 | 0.00 |
| | | 2013 | 255.51 | 0.00 | 0.00 | 18.03 | 0.00 | 0.00 |
| | | 2014 | 218.19 | 0.00 | 0.00 | 825.08 | 0.00 | 0.00 |
| | | 2015 | 141.88 | 0.00 | 0.00 | 31.49 | 0.00 | 0.00 |
| | | 2016 | 173.32 | 0.00 | 0.00 | 3.75 | 0.00 | 0.00 |
| | | 2017 | 191.64 | 0.00 | 0.00 | 2.77 | 0.00 | 0.00 |
| | | 2018 | 133.11 | 0.00 | 0.00 | 3.96 | 0.00 | 0.00 |
| | | 2019 | 67.12 | 0.00 | 0.00 | 1.37 | 0.00 | 0.00 |

*Results expressed in standard spray coverage ($10^6$ $m^2$)

**Bacterial larvicides, insect growth regulators and spinosyns

**Table S6**. Pyrethroid resistance versus pyrethroid use per country. Presented are the results per country (anonymized) per year. Confirmed resistance implies <90% mortality of anophelines in insecticide susceptibility bioassays for pyrethroids [3]; 0, no confirmed resistance; 1, confirmed resistance. Pyrethroids in use refers to the use in IRS for malaria control; 0, no pyrethroids in use; 1, pyrethroids in use.

| Year | Country code | Confirmed resistance | Pyrethroids in use |
|---|---|---|---|
| 2010 | 9 | 1 | 0 |
| 2010 | 13 | 0 | 1 |
| 2010 | 14 | 1 | 0 |
| 2010 | 19 | 1 | 1 |
| 2010 | 30 | 1 | 0 |
| 2010 | 32 | 1 | 1 |
| 2010 | 35 | 1 | 1 |
| 2010 | 40 | 0 | 1 |
| 2010 | 42 | 0 | 1 |
| 2010 | 50 | 0 | 1 |
| 2010 | 52 | 1 | 1 |
| 2010 | 58 | 0 | 1 |
| 2010 | 60 | 0 | 1 |
| 2010 | 69 | 0 | 1 |
| 2010 | 71 | 1 | 1 |
| 2010 | 74 | 0 | 1 |
| 2010 | 75 | 1 | 1 |
| 2010 | 77 | 1 | 1 |
| 2010 | 86 | 0 | 1 |
| 2010 | 87 | 0 | 1 |
| 2011 | 9 | 1 | 0 |
| 2011 | 11 | 0 | 1 |
| 2011 | 13 | 0 | 1 |
| 2011 | 14 | 1 | 0 |
| 2011 | 32 | 1 | 1 |
| 2011 | 35 | 1 | 1 |
| 2011 | 50 | 1 | 1 |
| 2011 | 59 | 0 | 1 |
| 2011 | 60 | 0 | 1 |
| 2011 | 65 | 1 | 1 |
| 2011 | 69 | 0 | 1 |
| 2011 | 71 | 1 | 1 |
| 2011 | 74 | 0 | 1 |
| 2011 | 75 | 1 | 1 |
| 2011 | 77 | 1 | 1 |
| 2011 | 83 | 1 | 0 |
| 2011 | 86 | 0 | 1 |
| 2011 | 88 | 1 | 1 |

| | | | |
|---|---|---|---|
| 2011 | 89 | 0 | 1 |
| 2012 | 9 | 1 | 0 |
| 2012 | 14 | 1 | 0 |
| 2012 | 19 | 1 | 1 |
| 2012 | 32 | 1 | 1 |
| 2012 | 35 | 1 | 1 |
| 2012 | 40 | 0 | 1 |
| 2012 | 42 | 0 | 1 |
| 2012 | 50 | 0 | 1 |
| 2012 | 52 | 1 | 0 |
| 2012 | 58 | 0 | 1 |
| 2012 | 59 | 0 | 1 |
| 2012 | 60 | 0 | 1 |
| 2012 | 65 | 1 | 1 |
| 2012 | 69 | 0 | 1 |
| 2012 | 70 | 0 | 1 |
| 2012 | 71 | 1 | 0 |
| 2012 | 74 | 0 | 1 |
| 2012 | 75 | 1 | 0 |
| 2012 | 77 | 1 | 1 |
| 2012 | 83 | 1 | 0 |
| 2012 | 86 | 0 | 1 |
| 2012 | 87 | 0 | 1 |
| 2012 | 88 | 1 | 1 |
| 2012 | 89 | 0 | 1 |
| 2013 | 9 | 0 | 0 |
| 2013 | 13 | 0 | 1 |
| 2013 | 19 | 1 | 1 |
| 2013 | 30 | 0 | 0 |
| 2013 | 32 | 1 | 1 |
| 2013 | 35 | 1 | 0 |
| 2013 | 40 | 1 | 1 |
| 2013 | 42 | 0 | 1 |
| 2013 | 50 | 0 | 1 |
| 2013 | 52 | 1 | 0 |
| 2013 | 58 | 0 | 1 |
| 2013 | 59 | 0 | 0 |
| 2013 | 60 | 0 | 1 |
| 2013 | 65 | 1 | 1 |
| 2013 | 69 | 1 | 1 |
| 2013 | 71 | 1 | 0 |
| 2013 | 75 | 1 | 1 |
| 2013 | 77 | 1 | 1 |
| 2013 | 78 | 0 | 1 |

| | | | |
|---|---|---|---|
| 2013 | 83 | 1 | 0 |
| 2013 | 86 | 0 | 1 |
| 2013 | 87 | 1 | 1 |
| 2013 | 88 | 1 | 0 |
| 2013 | 89 | 1 | 1 |
| 2014 | 9 | 1 | 0 |
| 2014 | 13 | 1 | 1 |
| 2014 | 19 | 1 | 1 |
| 2014 | 22 | 1 | 0 |
| 2014 | 30 | 1 | 1 |
| 2014 | 32 | 1 | 0 |
| 2014 | 35 | 1 | 0 |
| 2014 | 40 | 0 | 1 |
| 2014 | 42 | 0 | 1 |
| 2014 | 50 | 0 | 1 |
| 2014 | 58 | 0 | 1 |
| 2014 | 59 | 0 | 1 |
| 2014 | 60 | 0 | 1 |
| 2014 | 65 | 1 | 1 |
| 2014 | 71 | 1 | 0 |
| 2014 | 74 | 0 | 1 |
| 2014 | 75 | 1 | 1 |
| 2014 | 77 | 1 | 0 |
| 2014 | 78 | 1 | 1 |
| 2014 | 86 | 0 | 1 |
| 2014 | 87 | 1 | 1 |
| 2014 | 88 | 1 | 0 |
| 2014 | 89 | 1 | 1 |
| 2014 | 91 | 0 | 1 |
| 2015 | 9 | 1 | 0 |
| 2015 | 11 | 0 | 1 |
| 2015 | 19 | 1 | 1 |
| 2015 | 32 | 1 | 0 |
| 2015 | 35 | 1 | 0 |
| 2015 | 40 | 1 | 1 |
| 2015 | 42 | 0 | 1 |
| 2015 | 50 | 0 | 1 |
| 2015 | 52 | 1 | 0 |
| 2015 | 58 | 0 | 1 |
| 2015 | 59 | 0 | 1 |
| 2015 | 69 | 1 | 0 |
| 2015 | 71 | 1 | 0 |
| 2015 | 74 | 0 | 1 |
| 2015 | 75 | 1 | 1 |

| | | | |
|---|---|---|---|
| 2015 | 77 | 1 | 0 |
| 2015 | 78 | 0 | 1 |
| 2015 | 83 | 1 | 0 |
| 2015 | 86 | 0 | 1 |
| 2015 | 87 | 0 | 0 |
| 2015 | 88 | 1 | 0 |
| 2015 | 89 | 0 | 1 |
| 2015 | 91 | 1 | 1 |
| 2016 | 9 | 1 | 0 |
| 2016 | 19 | 1 | 1 |
| 2016 | 22 | 1 | 1 |
| 2016 | 30 | 1 | 1 |
| 2016 | 32 | 1 | 0 |
| 2016 | 35 | 1 | 0 |
| 2016 | 40 | 0 | 1 |
| 2016 | 52 | 1 | 0 |
| 2016 | 58 | 1 | 1 |
| 2016 | 59 | 1 | 1 |
| 2016 | 71 | 1 | 0 |
| 2016 | 74 | 0 | 1 |
| 2016 | 77 | 1 | 0 |
| 2016 | 83 | 1 | 0 |
| 2016 | 86 | 1 | 1 |
| 2016 | 87 | 1 | 0 |
| 2016 | 88 | 1 | 0 |
| 2016 | 91 | 1 | 1 |
| 2017 | 9 | 1 | 0 |
| 2017 | 13 | 1 | 1 |
| 2017 | 19 | 1 | 1 |
| 2017 | 35 | 1 | 0 |
| 2017 | 40 | 1 | 1 |
| 2017 | 50 | 1 | 0 |
| 2017 | 59 | 0 | 1 |
| 2017 | 65 | 1 | 1 |
| 2017 | 70 | 0 | 1 |
| 2017 | 75 | 1 | 1 |
| 2017 | 86 | 0 | 1 |
| 2017 | 87 | 1 | 0 |
| 2017 | 88 | 1 | 0 |
| 2018 | 13 | 1 | 1 |
| 2018 | 14 | 1 | 0 |
| 2018 | 19 | 1 | 1 |
| 2018 | 32 | 1 | 0 |
| 2018 | 40 | 0 | 1 |

| | | | |
|---|---|---|---|
| 2018 | 59 | 0 | 1 |
| 2018 | 60 | 0 | 1 |
| 2018 | 65 | 1 | 1 |
| 2018 | 74 | 0 | 1 |
| 2018 | 87 | 1 | 0 |
| 2019 | 11 | 0 | 1 |
| 2019 | 13 | 1 | 0 |
| 2019 | 30 | 1 | 0 |
| 2019 | 32 | 1 | 0 |
| 2019 | 35 | 1 | 0 |
| 2019 | 40 | 1 | 1 |
| 2019 | 52 | 1 | 0 |
| 2019 | 74 | 0 | 1 |
| 2019 | 87 | 1 | 0 |

**Table S7**. Degree of insecticide resistance management for disease vector control. Values are shown of parameter *R* and its components of rotation, mosaic/combination spraying, and multiplicity of modes of action, per disease for individual eligible countries (anonymized) during two five-year periods.

| | | | Period 1: 2010–2014 | | | | Period 2: 2015–2019 | | | |
|---|---|---|---|---|---|---|---|---|---|---|
| Disease | Country code | Region* | Rotation (*a*) | Mosaic (*b*-1) | Multiplicity (*c*-1) | Total (*R*) | Rotation (*a*) | Mosaic (*b*-1) | Multiplicity (*c*-1) | Total (*R*) |
| Malaria | 9 | AF | 1.00 | 0.10 | 0.50 | 1.60 | 0.00 | 0.00 | 0.00 | 0.00 |
| | 13 | AF | 1.00 | 0.40 | 0.50 | 1.90 | 3.00 | 0.30 | 1.50 | 4.80 |
| | 25 | AF | 1.00 | 0.60 | 1.00 | 2.60 | 2.00 | 0.60 | 2.00 | 4.60 |
| | 30 | AF | 3.00 | 0.29 | 1.50 | 4.79 | 2.50 | 0.17 | 1.00 | 3.67 |
| | 31 | AF | 0.00 | 0.50 | 0.50 | 1.00 | 5.50 | 0.30 | 2.50 | 8.30 |
| | 32 | AF | 2.00 | 0.60 | 1.00 | 3.60 | 1.00 | 0.13 | 1.00 | 2.13 |
| | 35 | AF | 2.00 | 0.20 | 1.00 | 3.20 | 1.00 | 0.25 | 1.00 | 2.25 |
| | 50 | AF | 0.50 | 1.20 | 1.50 | 3.20 | 2.50 | 0.63 | 2.50 | 5.63 |
| | 52 | AF | 2.50 | 0.10 | 1.50 | 4.10 | 1.00 | 0.25 | 1.00 | 2.25 |
| | 58 | AF | 4.00 | 1.00 | 1.50 | 6.50 | 3.50 | 1.10 | 2.00 | 6.60 |
| | 60 | AF | 0.00 | 0.50 | 0.50 | 1.00 | 1.50 | 0.80 | 1.50 | 3.80 |
| | 69 | AF | 1.00 | 0.40 | 1.00 | 2.40 | 1.00 | 0.13 | 0.50 | 1.63 |
| | 91 | AF | 2.00 | 0.70 | 1.50 | 4.20 | 0.00 | 0.50 | 0.50 | 1.00 |
| | 77 | AF | 3.00 | 0.40 | 1.50 | 4.90 | 1.00 | 0.25 | 1.00 | 2.25 |
| | 83 | AF | 0.00 | 0.00 | 0.00 | 0.00 | 1.00 | 0.25 | 0.50 | 1.75 |
| | 88 | AF | 1.00 | 1.30 | 2.00 | 4.30 | 3.00 | 0.40 | 2.00 | 5.40 |
| | 89 | AF | 0.00 | 1.10 | 0.50 | 1.60 | 1.00 | 2.00 | 2.50 | 5.50 |
| | 92 | LA | 0.00 | 0.00 | 0.00 | 0.00 | 0.00 | 0.00 | 0.00 | 0.00 |
| | 8 | LA | 0.00 | 1.00 | 1.00 | 2.00 | 0.00 | 1.00 | 1.00 | 2.00 |
| | 11 | LA | 1.00 | 0.20 | 1.00 | 2.20 | 0.00 | 1.00 | 1.00 | 2.00 |
| | 18 | LA | 0.00 | 0.00 | 0.00 | 0.00 | 2.00 | 0.00 | 1.00 | 3.00 |
| | 21 | LA | 0.00 | 0.00 | 0.00 | 0.00 | 0.00 | 0.00 | 0.00 | 0.00 |
| | 37 | LA | 0.00 | 0.00 | 0.00 | 0.00 | 0.00 | 0.00 | 0.00 | 0.00 |
| | 67 | LA | 0.00 | 0.00 | 0.00 | 0.00 | 0.00 | 0.00 | 0.00 | 0.00 |
| | 44 | AP | 0.00 | 0.00 | 0.00 | 0.00 | 0.00 | 0.00 | 0.00 | 0.00 |
| | 65 | AP | 0.00 | 0.00 | 0.00 | 0.00 | 0.00 | 0.00 | 0.00 | 0.00 |
| | 70 | AP | 0.00 | 0.00 | 0.00 | 0.00 | 0.00 | 0.00 | 0.00 | 0.00 |
| | 87 | AP | 1.00 | 0.40 | 1.00 | 2.40 | 1.00 | 0.20 | 2.00 | 3.20 |
| | 40 | AP | 0.00 | 1.50 | 1.50 | 3.00 | 0.00 | 1.50 | 1.50 | 3.00 |
| | 59 | AP | 4.00 | 0.50 | 1.50 | 6.00 | 0.00 | 0.00 | 0.00 | 0.00 |
| | 61 | AP | 0.00 | 0.00 | 0.00 | 0.00 | 0.00 | 0.00 | 0.00 | 0.00 |
| | 74 | AP | 1.00 | 0.60 | 1.00 | 2.60 | 0.00 | 0.00 | 0.00 | 0.00 |
| | 86 | AP | 0.00 | 0.00 | 0.00 | 0.00 | 0.00 | 0.00 | 0.00 | 0.00 |
| Dengue | 92 | LA | 1.00 | 0.40 | 0.50 | 1.90 | 0.50 | 0.40 | 0.50 | 1.40 |
| | 8 | LA | 0.00 | 1.00 | 1.00 | 2.00 | 0.00 | 1.00 | 1.00 | 2.00 |
| | 21 | LA | 0.00 | 0.00 | 0.00 | 0.00 | 0.00 | 0.00 | 0.00 | 0.00 |
| | 23 | LA | 0.00 | 1.50 | 1.50 | 3.00 | 0.00 | 1.50 | 1.50 | 3.00 |
| | 37 | LA | 0.00 | 0.00 | 0.00 | 0.00 | 0.00 | 0.00 | 0.00 | 0.00 |

| | | | | | | | | | | |
|---|---|---|---|---|---|---|---|---|---|---|
| | 54 | LA | 0.00 | 1.50 | 1.50 | 3.00 | 1.00 | 2.10 | 2.50 | 5.60 |
| | 67 | LA | 0.00 | 0.00 | 0.00 | 0.00 | 0.00 | 1.00 | 1.00 | 2.00 |
| | 65 | AP | 0.00 | 0.00 | 0.00 | 0.00 | 0.00 | 0.00 | 0.00 | 0.00 |
| | 70 | AP | 0.00 | 0.00 | 0.00 | 0.00 | 0.00 | 0.00 | 0.00 | 0.00 |
| | 40 | AP | 0.00 | 0.00 | 0.00 | 0.00 | 0.00 | 0.00 | 0.00 | 0.00 |
| | 41 | AP | 2.00 | 0.80 | 1.00 | 3.80 | 2.00 | 0.60 | 1.00 | 3.60 |
| | 74 | AP | 0.00 | 1.00 | 1.00 | 2.00 | 0.00 | 1.00 | 1.00 | 2.00 |
| | 33 | AP | 0.00 | 1.00 | 1.00 | 2.00 | 0.00 | 1.00 | 1.00 | 2.00 |
| | 19 | AP | 0.50 | 1.40 | 1.50 | 3.40 | 0.50 | 1.40 | 1.50 | 3.40 |
| Leishmaniasis | 43 | AP | 0.00 | 1.00 | 1.00 | 2.00 | 0.00 | 1.00 | 1.00 | 2.00 |
| | 70 | AP | 0.00 | 0.00 | 0.00 | 0.00 | 0.00 | 0.00 | 0.00 | 0.00 |
| | 76 | AP | 1.00 | 0.20 | 1.00 | 2.20 | 0.00 | 0.00 | 0.00 | 0.00 |
| | 7 | AP | 0.00 | 0.00 | 0.00 | 0.00 | 0.00 | 0.00 | 0.00 | 0.00 |
| | 61 | AP | 0.00 | 0.00 | 0.00 | 0.00 | 0.00 | 0.00 | 0.00 | 0.00 |
| Chagas disease | 92 | LA | 0.00 | 0.00 | 0.00 | 0.00 | 0.00 | 0.00 | 0.00 | 0.00 |
| | 11 | LA | 0.00 | 1.00 | 1.00 | 2.00 | 0.00 | 1.00 | 1.00 | 2.00 |
| | 18 | LA | 0.00 | 0.00 | 0.00 | 0.00 | 0.00 | 0.00 | 0.00 | 0.00 |

*AF, African; AP, Asia-Pacific; LA, Latin American & Caribbean

## References


1 Najera, J. A. & Zaim, M. *Malaria vector control: decision making criteria and procedures for judicious use of insecticides. WHO/CDS/WHOPES/2002.5 Rev.1*. (World Health Organization, 2002).

2 Rozendaal, J. A. *Vector control: methods for use by individuals and communities*. (World Health Organization, 1997).

3 WHO. *Malaria Threats Map. https://apps.who.int/malaria/maps/threats/ Accessed: 27 January 2021*, 2021).

4 WHO. *Test procedures for insecticide resistance monitoring in malaria vector mosquitoes – 2nd ed*. (World Health Organization, 2016).

5 UN. *United Nations Regional Groups of Member States. https://www.un.org/dgacm/en/content/regional-groups*, 2021).

6 UN. (Department of Economic and Social Affairs, Population Division, United Nations, New York, 2020).
